# Mechanical Framework for Geopolymer Gels Construction: An Optimized LSTM Technique to Predict Compressive Strength of Fly Ash-Based Geopolymer Gels Concrete

**DOI:** 10.3390/gels10020148

**Published:** 2024-02-16

**Authors:** Xuyang Shi, Shuzhao Chen, Qiang Wang, Yijun Lu, Shisong Ren, Jiandong Huang

**Affiliations:** 1 School of Mines, China University of Mining and Technology, Xuzhou 221116, China; shixuyangcumt@126.com (X.S.); wangqiang_cumt@163.com (Q.W.); 2 School of Civil Engineering, Guangzhou University, Guangzhou 510006, China; yijun.lu@e.gzhu.edu.cn; 3 Faculty of Civil Engineering and Geosciences, Delft University of Technology, 2628 Delft, The Netherlands

**Keywords:** long short-term memory networks, compressive strength, prediction, marine predators algorithm

## Abstract

As an environmentally responsible alternative to conventional concrete, geopolymer concrete recycles previously used resources to prepare the cementitious component of the product. The challenging issue with employing geopolymer concrete in the building business is the absence of a standard mix design. According to the chemical composition of its components, this work proposes a thorough system or framework for estimating the compressive strength of fly ash-based geopolymer concrete (FAGC). It could be possible to construct a system for predicting the compressive strength of FAGC using soft computing methods, thereby avoiding the requirement for time-consuming and expensive experimental tests. A complete database of 162 compressive strength datasets was gathered from the research papers that were published between the years 2000 and 2020 and prepared to develop proposed models. To address the relationships between inputs and output variables, long short-term memory networks were deployed. Notably, the proposed model was examined using several soft computing methods. The modeling process incorporated 17 variables that affect the CSFAG, such as percentage of SiO_2_ (SiO_2_), percentage of Na_2_O (Na_2_O), percentage of CaO (CaO), percentage of Al_2_O_3_ (Al_2_O_3_), percentage of Fe_2_O_3_ (Fe_2_O_3_), fly ash (FA), coarse aggregate (CAgg), fine aggregate (FAgg), Sodium Hydroxide solution (SH), Sodium Silicate solution (SS), extra water (EW), superplasticizer (SP), SH concentration, percentage of SiO_2_ in SS, percentage of Na_2_O in SS, curing time, curing temperature that the proposed model was examined to several soft computing methods such as multi-layer perception neural network (MLPNN), Bayesian regularized neural network (BRNN), generalized feed-forward neural networks (GFNN), support vector regression (SVR), decision tree (DT), random forest (RF), and LSTM. Three main innovations of this study are using the LSTM model for predicting FAGC, optimizing the LSTM model by a new evolutionary algorithm called the marine predators algorithm (MPA), and considering the six new inputs in the modeling process, such as aggregate to total mass ratio, fine aggregate to total aggregate mass ratio, FASiO_2_:Al_2_O_3_ molar ratio, FA SiO_2_:Fe_2_O_3_ molar ratio, AA Na_2_O:SiO_2_ molar ratio, and the sum of SiO_2_, Al_2_O_3_, and Fe_2_O_3_ percent in FA. The performance capacity of LSTM-MPA was evaluated with other artificial intelligence models. The results indicate that the R^2^ and RMSE values for the proposed LSTM-MPA model were as follows: MLPNN (R^2^ = 0.896, RMSE = 3.745), BRNN (R^2^ = 0.931, RMSE = 2.785), GFFNN (R^2^ = 0.926, RMSE = 2.926), SVR-L (R^2^ = 0.921, RMSE = 3.017), SVR-P (R^2^ = 0.920, RMSE = 3.291), SVR-S (R^2^ = 0.934, RMSE = 2.823), SVR-RBF (R^2^ = 0.916, RMSE = 3.114), DT (R^2^ = 0.934, RMSE = 2.711), RF (R^2^ = 0.938, RMSE = 2.892), LSTM (R^2^ = 0.9725, RMSE = 1.7816), LSTM-MPA (R^2^ = 0.9940, RMSE = 0.8332), and LSTM-PSO (R^2^ = 0.9804, RMSE = 1.5221). Therefore, the proposed LSTM-MPA model can be employed as a reliable and accurate model for predicting CSFAG. Noteworthy, the results demonstrated the significance and influence of fly ash and sodium silicate solution chemical compositions on the compressive strength of FAGC. These variables could adequately present variations in the best mix designs discovered in earlier investigations. The suggested approach may also save time and money by accurately estimating the compressive strength of FAGC with low calcium content.

## 1. Introduction

Due to the fast rise in the world’s population as well as the gradual degradation of the world’s infrastructure, there is an urgent requirement for the quick development of the construction sector. Ordinary Portland Cement (OPC) is a binder used frequently in concrete, one of the most common construction materials [1,2,3,4,5,6]. Using OPC-based concrete presents several environmental challenges, the most significant of which is the substantial quantity of cement that is required, which in turn results in an enormous amount of carbon dioxide (CO_2_) emissions. The calcination process and the use of energy to produce OPC both contribute to the production of CO_2_. About 5–7% of the total yearly CO_2_ emissions are attributed, according to previous research, to the production of OPC [7]. Hence, lowering OPC usage in the building sector reduces CO_2_ emissions, which may also slow the acceleration of climatic changes [8]. In an effort to reduce OPC-related CO_2_ emissions, several scientists have investigated using environmentally friendly binders, including fly ash (FA), in diverse building supplies involving brick-and-mortar [9] and compact soil walls [10]. Furthermore, geopolymer concrete, which is both structurally economical and environmentally beneficial, shall be properly explored to complement OPC concrete in the building sector.

When compared to OPC concrete, geopolymer concrete can reduce CO_2_ emissions by approximately 44–64% [11]. Davidovits presented geopolymer concretes in 1991 [12]. FA, used as a key precursor, is sourced from coal-fired power plants, where it is generated as a by-product of the combustion process. Specifically, the fly ash is obtained from thermal power plants utilizing coal as the primary fuel source. Besides reducing CO_2_ emissions, the use of FA in geopolymer concrete reduces additional ecological and pollution issues associated with the disposal of FA as waste. Over the past years, several scientists have examined the engineering specifications (mechanical and chemical) of fly ash-based geopolymer concrete (FAGC) and its design factors based on the aforementioned qualities. Previous analysis has demonstrated that FAGC has sufficient mechanical qualities, including compressive strength (CS), high durability against fire, and sulfate resistance [13,14,15,16].

Previous studies on the key parameters and their effects have been conducted. Hardjito and Rangan [14] highlighted that the water-to-binder material ratio decreases the compressive strength of FAGC (CSFAGC). In another study, the direct relationship between alkali activator liquid to FA material and the CSFAGC was demonstrated by Pavithra et al. [17]. In other studies, Al Bakri et al. [18] and Phoo-ngernkham et al. [19] stated that sodium silicate (SS) solution to sodium hydroxide (SH) solution mass ratio has a considerable impact on the CSFAGC. Moreover, Joseph and Mathew [20] and Al Bakri et al. [18], respectively, specified that the optimum SS solution to SH solution mass ratio was equal to 2 and 2.5. One of the other key parameters is the concentration of the SH solution. In this regard, Mustafa Al Bakri et al. [18] found that the concentration of SH solution was 12 M. Furthermore, the optimal SH solution concentration is 10 M; incrementing and decrementing the SH solution concentration from 10 M resulted in a decrease in the CSFAGC. Hardjito and Rangan [14] considered the key parameter of the ratio of superplasticizer to FA, whose value greater than 0.02 resulted in a decrease in the CSFAGC. In addition, they concluded that the use of a superplasticizer based on carboxylate resulted in a greater CS than that of a superplasticizer based on naphthalene. Curing time is another key parameter; the value of 24 h was obtained by Abdulkareem and Ramli [21] for FA paste. In other studies, Hardjito and Rangan [14] stated that increased curing time intensified the tendency to increase CS. The duration and type of curing for their test were various curing periods from 4 h to 96 h (4 days) and steam-curing, respectively; nevertheless, behind 24 h, the increasing rate decreased. Accordingly, the optimal curing time is 24 h. Curing temperature is another key parameter that affects CSFAGC, as Abdulkareem and Ramli [21] found out that enhancing the curing temperature to seventy degrees Celsius increases the CS. In addition, Joseph and Mathew [20] and Hardjito and Rangan [14] highlighted that increasing the curing temperature increases the CSFAGC. Tang et al. [22] studied the CS behaviors of fly ash/slag-based geopolymeric concretes with recycled aggregate.

In recent investigations, sodium- and potassium-based alkali activator (AA) solutions were utilized. Due to the qualitative nature of the type of AA parameter and since the regression approach is only capable of discovering the formalization of quantitative parameters, in order to accomplish the modeling by means of a statistical model, it is necessary to take into consideration at least one form of AA solution. According to the findings of Chau-Khun Ma et al. [23], the combining of SS and SH solutions was the most common source of AA in previously conducted investigations.

Based on a study conducted by Assi et al. [24], the sources of FA greatly impact the CSFAGC. De Silva et al. [25] noticed that enhancing the ratio of SiO_2_ to Al_2_O_3_ improved the CSFAGC. Furthermore, based on research performed by Davidovits [26], Fe_2_O_3_ contributes to geo-polymerization processes. The incorporation of Fe_2_O_3_ in the geo-polymerization processes of geopolymer concretes can have several notable effects on the properties and performance of the resulting material. Fe_2_O_3_, when added to geopolymer concretes, has been reported to contribute to increased compressive strength and durability. The presence of Fe_2_O_3_ can promote the formation of a more compact and stable geopolymeric matrix, resulting in improved mechanical properties. Furthermore, it imparts a reddish-brown color to geopolymer concretes. This characteristic coloration can be aesthetically pleasing and is often preferred for certain architectural or decorative applications. Notably, the addition of Fe_2_O_3_ can affect the setting time and workability of geopolymer concretes. Careful consideration must be given to the dosage of Fe_2_O_3_ to avoid potential challenges related to accelerated or delayed setting times and alterations in the workability of the mix. It can influence the microstructure of geopolymer concretes by participating in the geo-polymerization reactions. This can result in changes to the pore size distribution, leading to a more refined and homogeneous microstructure. Geopolymers containing Fe_2_O_3_ may exhibit enhanced thermal stability and resistance. This can be attributed to the influence of iron oxide on the geopolymeric network, leading to improved resistance to high temperatures and fire. Geopolymers incorporating Fe_2_O_3_ may demonstrate improved resistance to certain chemical attacks. The presence of iron oxide can contribute to a denser and less porous structure, enhancing the material’s resistance to chemical aggression. Moreover, the use of Fe_2_O_3_ in geopolymer concrete aligns with sustainable practices as it provides an opportunity to utilize industrial by-products or waste materials containing iron oxide. This contributes to the environmentally friendly nature of geopolymer technology [27].

It is crucial to note that the specific effects of Fe_2_O_3_ can depend on factors such as its concentration, the overall mix design, and the curing conditions [28,29]. Consequently, a detailed understanding of these aspects is essential for optimizing the performance of geopolymer concretes incorporating Fe_2_O_3_. However, according to ASTM C618-19, the total of the percentages of SiO_2_, Al_2_O_3_, and Fe_2_O_3_ in FA is one of the primary features of class F FA [16,30,31,32]. Consequently, it could be deduced that the SiO_2_, Al_2_O_3_, and Fe_2_O_3_ compositions of FA influence the CSFAGC.

**Figure 1:**
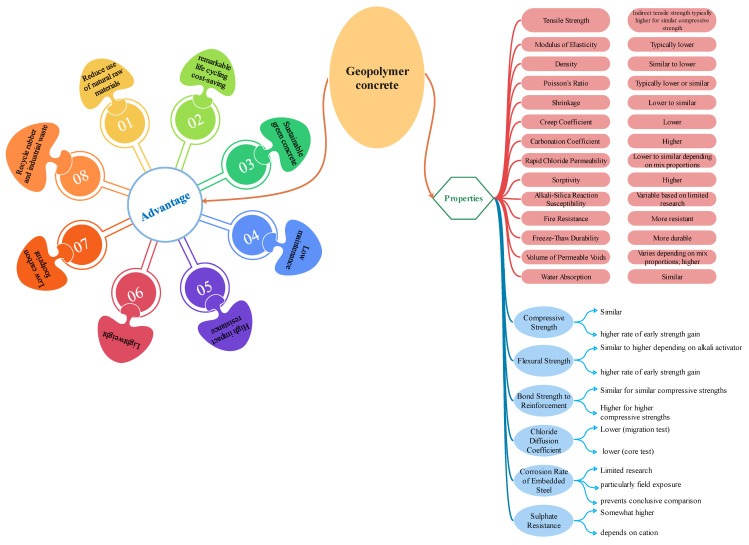
Advantages and properties of geopolymer concrete.

Non-dimensional factors, including the SiO_2_:Al_2_O_3_ molar ratio in FA [33], the Na_2_O:SiO_2_ molar ratio in FA [33], the H_2_O:Na_2_O molar ratio in FA, and the Fe_2_O_3_:SiO_2_ molar ratio in FA, may be viewed as the essential factors of the CSFAGC. Even though the majority of studies have examined the impact of the SS solution to SH solution mass ratio on the CSFAGC, a few studies [34,35,36,37] revealed that the crucial factors in the alkali activator liquid were the SiO_2_:Na_2_O and H_2_O:Na_2_O molar ratios. These characteristics may account for variations in the SS solution and SH solution chemical compositions. Contrary to these findings, the majority of studies have solely used the mass of FAGC elements as predictor factors when proposing a framework for the CSFAGC. The main advantages and properties of concrete are listed in Figure 1.

The estimation of required results is now commonly performed using machine learning (ML) techniques. Using a variety of ML approaches, concrete’s mechanical characteristics may be accurately predicted. To estimate various characteristics of concretes, several techniques such as artificial neural networks (ANNs), support vector machines (SVMs), gene expression programming (GEP), decision trees (DTs), and ensemble ML techniques were widely utilized by researchers [27,38,39,40,41,42,43,44,45,46,47,48,49,50,51,52]. The GEP approach utilized by Iqbal et al. [53] for forecasting the mechanical specifications of eco-friendly concretes, including waste foundry sand, serves as an example of the model’s successful application to prediction. The research of Golafshani et al. [54] used the ANNs technique to anticipate the mechanical characteristics of sustainable concrete made from foundry sand. According to research, any kind of concrete may be accurately predicted using the ANN approach. In order to forecast mechanical characteristics from microstructure pictures in the fiber-reinforced polymer, Sun et al. [55] employed a typical neural network. They said that one might locate a likely damage location in a fiber-reinforced polymer using trained models. The capability of ANNs and SVR models in predicting the CS of concrete was compared in Akande et al.’s [56] research. The research indicated that the progress accuracy level of the SVR approach was marginally superior to the ANN technique. Table 1 summarizes the various ML techniques that have been used to estimate and model different properties of concrete incorporating different industry wastes.

This research aims to establish a robust predictive framework for the CSFAGC by using the chemical compositions of both FA and SS solutions. The dataset was gathered from articles that were published between 2000 and 2020 and included sodium-based alkali activator liquid in their mix designs.

The emphasis and innovation of the present study are threefold. First and foremost, this research intends to challenge how well network-based (MLPNN, GFFNN, and BRNN) models perform. Second, in order to estimate the CSFAGC, this study also assesses and employs metaheuristic algorithms (PSO and MPA) to optimize the LSTM model and make a more accurate estimation of the CSFAGC. Thirdly, the models consider six additional factors, including the aggregate to total mass ratio, fine aggregate to total aggregate mass ratio, FASiO_2_:Al_2_O_3_ molar ratio, FA SiO_2_:Fe_2_O_3_ molar ratio, AA Na_2_O:SiO_2_ molar ratio, and the sum of SiO_2_, Al_2_O_3_, and Fe_2_O_3_ percent in FA. According to the best of our knowledge, there is not a study like it that uses metaheuristic algorithm-based LSTM modeling for the CSFAGC in the literature. There is a lack of research in the literature that measures the impact of a number of different mixture percentage factors and various curing regimes on the compressive strength of FAGC. The authorized and established model that employed many characteristics to estimate the compressive strength of FAGC is frequently utilized in the construction industry, according to the comprehensive and systematic review that was conducted on FAGC [84,85,86]. This was found as a result of the research that was conducted on the FAGC. The greatest number of attempts have been focused on a single-scale model, and they have not included a wide range of laboratory test data or several factors. Additionally, the compressive strength of FAGC may be affected by multiple variables at a time. Hence, in this work, for the first time in a single constructed model, the effects of 17 factors were explored and evaluated on the compressive strength of FAGC. These factors include the percentage of SiO_2_ (SiO_2_), percentage of Na_2_O (Na_2_O), percentage of CaO (CaO), percentage of Al_2_O_3_ (Al_2_O_3_), percentage of Fe_2_O_3_ (Fe_2_O_3_), FA, coarse aggregate (CAgg), fine aggregate (FAgg), SH, SS, extra water (EW), superplasticizer (SP), SH concentration, percentage of SiO_2_ in SS, percentage of Na_2_O in SS, curing time, curing temperature, and CS. We employed various model methodologies, including MLPNN, BRNN, GFFNN, SVR-L, SVR-P, SVR-S, SVR-RBF, DT, RF, LSTM, and hybrid LSTM-PSO and LSTM-MPA. These models were tested using 162 samples gathered from the research previously performed; they had to be tested through their paces as prediction models in order to estimate the compressive strength of FAGC.

It is essential that the compressive strength values of geopolymer concrete be estimated under both mixed and simple circumstances in a manner that is either exact or close to their actual capability. If the appropriate primary theories have influential factors, and those factors are accessible for identical objectives, then the related principal theories can potentially be fundamentally applied. The majority of the time, empirical methods are viable alternatives; nevertheless, the process of configuring them and putting them into practice may be time-intensive. It may be difficult to generalize these empirical formulas to different situations because of the limited test data and the significant number of side parameters that were considered. This needs the structuring of a novel and accurate approach for precisely modeling and estimating the compressive strength of geopolymer concretes, taking into consideration the influence that varied combination proportions and curing times could have on the final product’s properties. Table 2 shows the studies that use a large number of parameters affecting the compressive strength of geopolymer concretes.

The studies that were addressed above showed that machine learning techniques could produce outstanding results with a greater number of parameters while overcoming limitations such as an inadequate amount of experimental data and an inability to generalize the model to other settings. There are many attempts to predict the compressive strength of geopolymer concretes. Ahmed et al. [100] obtained 250 laboratory samples to analyze the impacts of adding different dosages of nanosilica on compressive strength, splitting tensile strength, flexural strength, stress–strain behaviors, modulus of elasticity, water absorption, and rapid chloride permeability of geopolymer concrete composites. In another study, Ahmed et al. [101] used ANN multi-expression programming, full quadratic, linear regression, and M5P-tree to estimate the compressive strength of geopolymer concretes. Qaidi et al. [102] studied the printing process of three-dimensional printing technology for building applications utilizing geopolymers as suitable concrete materials. Compressive strength of geopolymer concretes prediction was performed by Ahmed et al. [103]. They used SVR techniques and optimized them with particle swarm optimization (PSO), support vector regression (SVR), grey wolf optimization (GWO), differential evolution (DE), and manta ray foraging optimization (MRFO) algorithms. They achieved acceptable results in predicting the compressive strength of geopolymer concretes. Ahmed et al. [104,105] established a predictive model based on the ANN, M5P, linear regression (LR), and multi-logistic regression (MLR) methods for predicting the compressive strength of geopolymer concretes. In another paper, Ahmed et al. [106] predicted CSFAGC by utilizing LR and MLR. In another study, Tanyildizi [91] used LSTM, KNN, and SVR techniques to predict the geo-polymerization process of fly ash-based geopolymers.

Because there has not been much research regarding how to estimate the compressive strength of geopolymer concretes with innovative parameters used to determine the comprehensive prediction model, such as aggregate to total mass ratio, fine aggregate to total aggregate mass ratio, FASiO_2_:Al_2_O_3_ molar ratio, FA SiO_2_:Fe_2_O_3_ molar ratio, AA Na_2_O:SiO_2_ molar ratio, and the sum of SiO_2_, Al_2_O_3_, and Fe_2_O_3_ percent in FA. Despite the fact that geopolymer concrete is employed in numerous initiatives, it is difficult to utilize them very swiftly in the construction industries. In addition, the construction industry is seeking increasing opportunities for innovative building supplies that have distinctive features and could improve the useful life of concrete buildings. Notable is the fact that the compressive strengths of geopolymer concretes vary depending on the different conditions, composite materials, and types of mixtures that are used. As a result, many parameters affect the compressive strengths of geopolymer concretes. This means that innovative systems need to be developed in order to anticipate the behavior of these novel materials.

## 2. Results and Discussion

The achieved results of the developed LSTM-MPA model are analyzed in this section. Notably, the ability of the MPA algorithm in the LSTM technique is shown by comparing the results of LSTM-MPA with conventional LSTM.

### 2.1. LSTM

One of the most fundamental components of artificial intelligence and soft computing techniques is hyperparameters, which have a considerable impact on the accuracy of models. According to recent studies, the efficiency of the LSTM hyperparameters can be comparable to that of the highly sophisticated LSTM approach, provided they are properly tuned and optimized [107,108,109]. The computational behavior of the optimized LSTM model, which uses a search to find the best global solutions in the search space, is its most significant benefit. The hyperparameter of LSTM models is optimized in the current study using MPA and PSO to estimate CSFAGC.

Therefore, we first developed the conventional LSTM model. Meanwhile, we manually set the hyperparameters of models, i.e., using a trial-and-error approach. It is obvious that endless models must be built in order to obtain an ideal model if we wish to choose the number and types of LSTM hyperparameters manually. Like most research articles, this takes a lot of time and is virtually impractical; thus, a low number of architectures were manually constructed by varying the values and types of hyperparameters.

**Figure 2:**
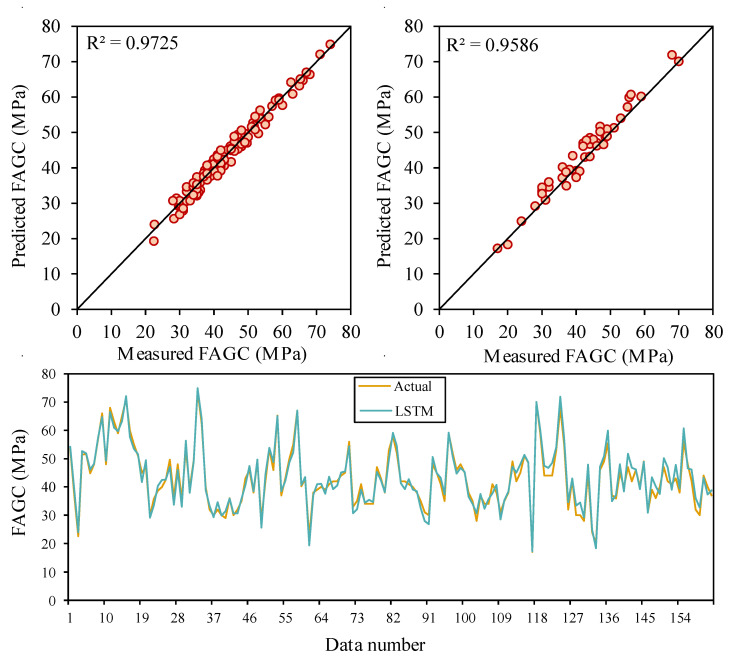
The correlation between measured and estimated CSFAGC values during training and testing of the LSTM model.

The LSTM technique is known as a randomness approach; therefore, the results can involve various predictions for each run. Therefore, the training process of the LSTM model is performed randomly for 45 runs, and hyperparameters are adjusted. As aforementioned, the performance level of models is considered employing the statistical indices, i.e., R^2^, Adjusted R^2^, MAE, MAPE, NS, RMSE, VAF, WI, WMAPE, MRE, PI, Bias, SI, and p. After the initial attempt, the specific range was determined for the hyperparameters of the LSTM model; in fact, the LSTM model is developed by various activation functions, batch size, epoch number, dropout rate, and hidden neurons. The range or type of these hyperparameters were as follows: epoch number [5–500], hidden neurons [5–150], batch size [5–150], dropout rate [0.1–0.5], and activation functions {exponential, linear, tanh, SELU, hard sigmoid, ReLu}. Finally, after evaluating the results of 45 various LSTM models, the optimal hyperparameters were specified by the trial-and-error procedure as the number of epochs of 150, the number of hidden neurons of 33, batch sizes of 17, the dropout rate of 0.3, and the activation function of the hard ReLu. The graph of predicted CSFAGC using the LSTM model compared to measured values of the train and test parts is illustrated in Figure 2. As shown in this graph, the LSTM model can provide a high-performance level in the estimation of CSFAGC. This model with these characteristics yielded a value of 0.9725 for the training phase and 0.9586 for the testing phase.

The achieved result for the manually adjusted LSTM predictive model shows that the performance of prediction and its accuracy are acceptable; however, structuring an LSTM model with this accuracy is prone to spending a lot of time, and the user faces unintentional problems and errors. Notably, only one model is selected as the one with the optimal structure among the obtained models. Meanwhile, there may be a structure that has different characteristics and hyperparameters compared to other achieved models and, at the same time, has higher accuracy. Therefore, it is very difficult and time-consuming to find the optimal structure manually.

The optimized LSTM-PSO and LSTM-MPA models automatically determine the types and values of hyperparameters regarding the LSTM model by the PSO and MPA algorithms. Therefore, the optimal structure with the optimum value for hyperparameters is obtained in a short time. In this study, the PSO algorithm was combined with the LSTM method to show the accuracy of the LSTM-MPA developed model. Moreover, the high capability of the LSTM was compared with six other methods, i.e., MLPNN, BRNN, GFFNN, SVR, DT, and RF.

### 2.2. LSTM-PSO

**Figure 3:**
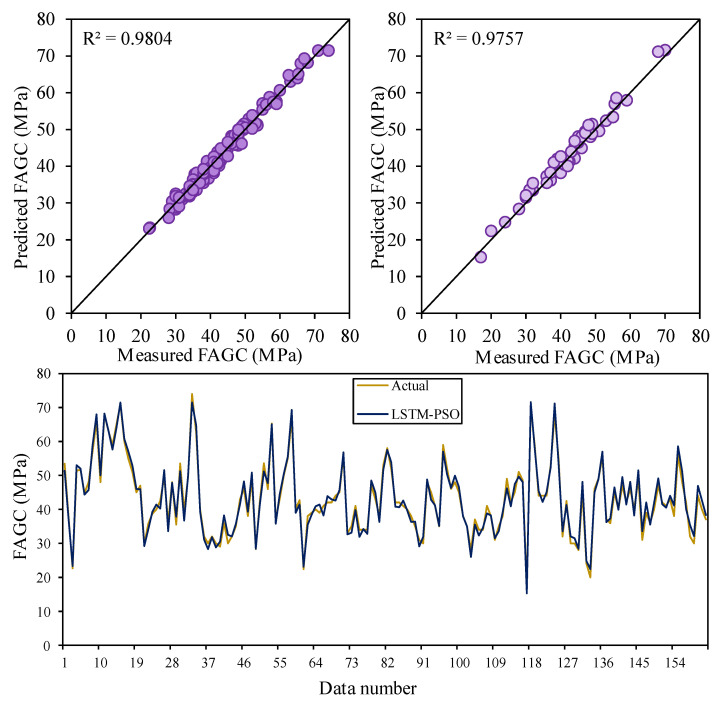
The correlation between measured and estimated CSFAGC values during training and testing the LSTM-PSO model.

**Figure 4:**
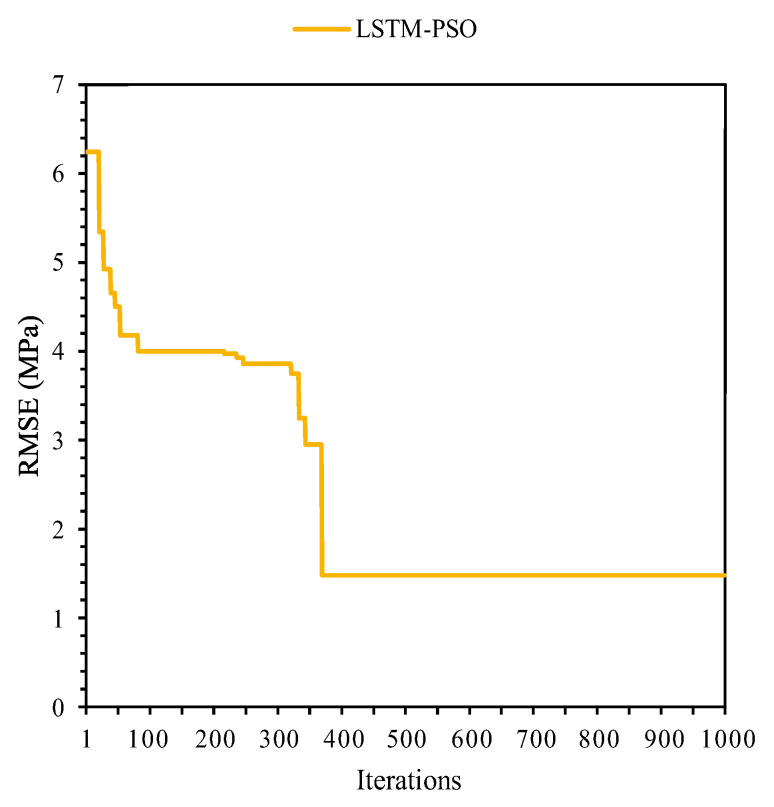
Iterative performance of the LSTM-PSO.

The LSTM-PSO model presents a fast convergence speed, minimum errors, and a good level of accuracy during the optimization phase. The optimization of the objective function (RMSE) in the PSO algorithm for obtaining the optimal values of hyperparameters in LSTM, as well as increasing the accuracy of structure, is depicted in Figure 3. As shown, the convergence graph shows that the LSTM-PSO has an acceptable effect. LSTM-PSO results revealed that the optimized values for the hyperparameters were: number of epochs = 45, number of hidden neurons = 29, batch size = 19, dropout rate = 0.15, activation function is hard sigmoid, number of boosting rounds = 57, eta = 0.199. The capabilities of LSTM-PSO in the prediction of the target are displayed in Figure 4, in which the measured and predicted values are compared and the accuracy of the LSTM-PSO model is evaluated. The obtained results indicated that the R^2^ values for the train and test phases of the LSTM-PSO model are 0.9804 and 0.9757, respectively. Therefore, this model is capable of estimating CSFAGC with a high level of accuracy and an acceptable degree of performance. Furthermore, the results of the LSTM-PSO model indicate that the performance of the developed LSTM-PSO is significantly higher than the manually optimized LSTM model.

### 2.3. LSTM-MPA

**Figure 5:**
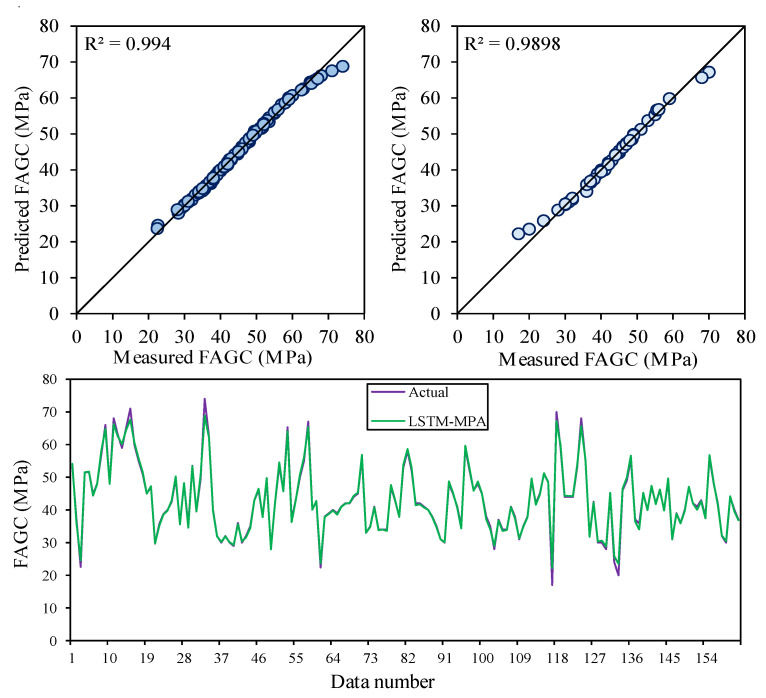
The correlation between measured and estimated CSFAGC values during training and testing of the LSTM-MPA model.

**Figure 6:**
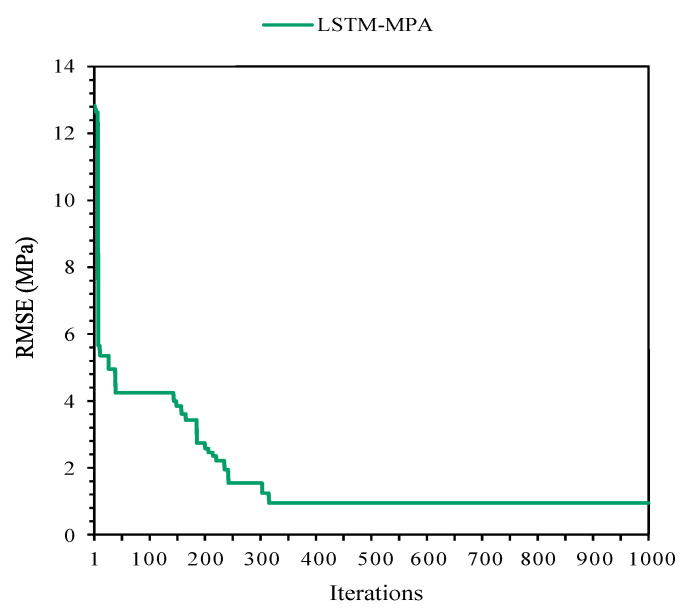
Iterative performance of the LSTM-MPA.

As mentioned before, achieving the most accurate LSTM predictive model is difficult. In this regard, the LSTM-MPA algorithm was established as a hybrid method to predict CSFAGC. The MPA is one of the newest and fastest optimization methods utilized to predict targets and solve optimization and engineering issues. Therefore, the combined MPA with LSTM approach is presented for predicting the CSFAGC herein. First, the parameters of the MPA algorithms were adjusted, and then the termination criterion was set. In this study, the termination criterion was the maximum number of iterations. Finally, a run of hybrid MPA-LSTM was performed, and the optimum hyperparameters were determined as the number of epochs = 75, the number of hidden neurons = 33, batch size = 12, dropout rate = 0.55, the activation function is hard sigmoid, the number of boosting rounds = 48, and eta = 0.355. The convergence of the RMSE value for LSTM-MPA is demonstrated in Figure 5. The R^2^ values of this model were, respectively, 0.9940 and 0.9898 for the training and testing portions. It can be concluded that the performance of LSTM-MPA is higher than that of LSTM and LSTM-PSO. Therefore, the LSTM-MPA is capable of predicting CSFAGC with the highest degree of accuracy. The measured and predicted CSFAGC by the proposed hybrid LSTM-MPA model is depicted in Figure 6.

### 2.4. Comparison of the Proposed Model with Other AI Models

**Figure 7:**
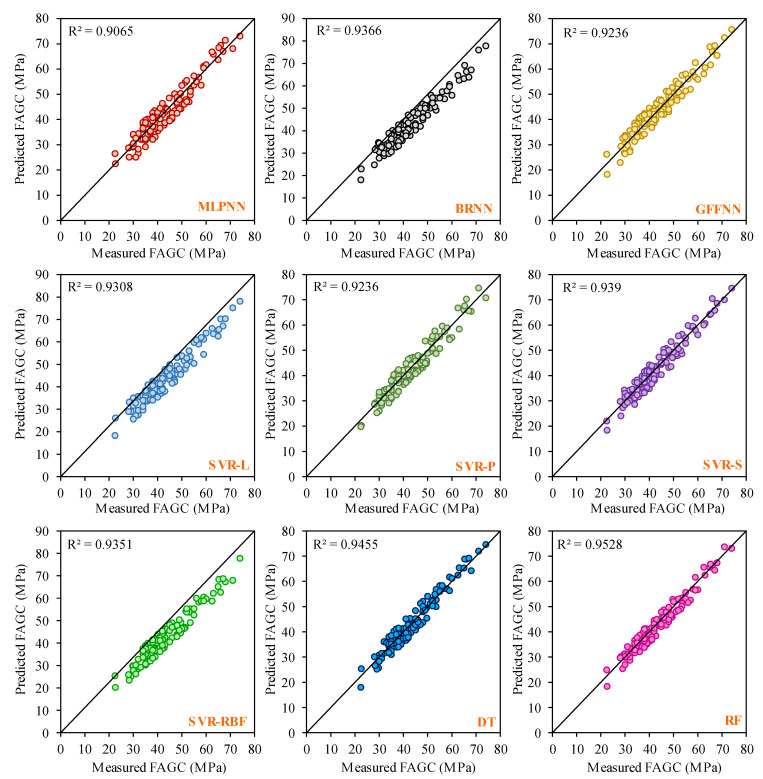
Correlation plot between actual and estimated values of the training parts of the ANN, SVR, DT, and RF models.

**Figure 8:**
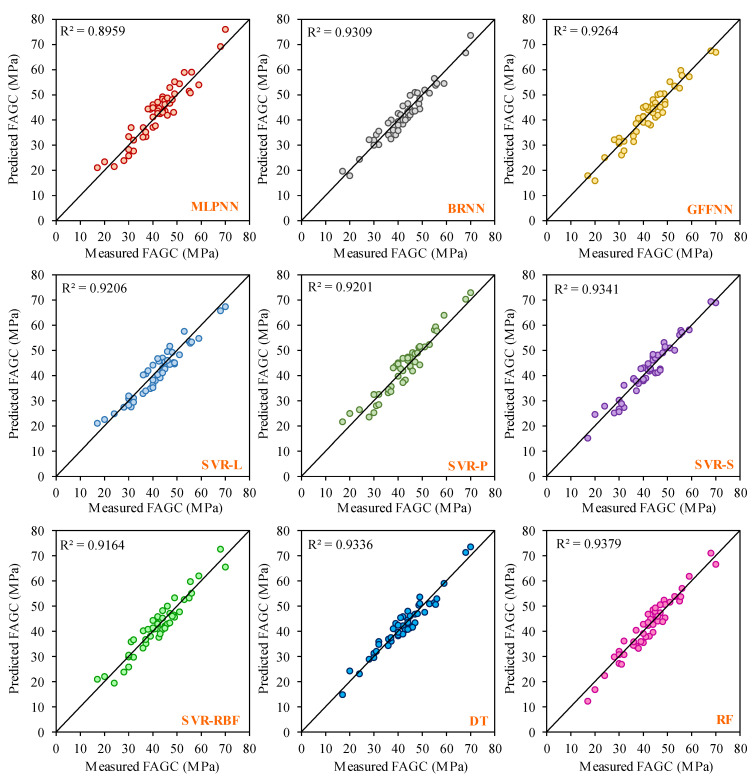
Correlation plot between actual and estimated values of testing parts of the ANN, SVR, DT, and RF models.

It can be valuable to compare the CSFAGC estimation outcomes obtained by the LSTM-MPA structure in the present study with those achieved by the other AI techniques. In this study, four additional AI techniques involving ANNs, SVR, DT, and RF were employed for predicting the CSFAGC in addition to the LSTM, LSTM-PSO, and LSTM-MPA models. These four models, like the LSTM model, were run 45 times, and the better model result was chosen from the developed models. Applying the statistical indices of Adj. R^2^, MAE, MAPE, NS, R, R^2^, RMSE, VAF, WI, WMAPE, PI, Bias, SI, p, and MRE to assess these models’ performance capacity to forecast the CSFAGC, it has resulted that their performance is suitable; however, their accuracy level is less than that of LSTM, LSTM-PSO, and LSTM-models. In the following, the prediction results of three types of ANNs, i.e., MLPNN, BRNN, and GFFNN, were presented. Furthermore, the SVR model with the four different kernel functions, i.e., sigmoid, linear, radial basis function, and polynomial, was developed. Therefore, the LSTM, LSTM-PSO, and LSTM-MPA models were compared to nine other models. For comparing and analyzing the predictor accuracy level of the proposed models, Table 3, Table 4, Table 5 and Table 6 and Figure 7 and Figure 8 reveal the performance, error, and accuracy of predictive models. The calculated statistical indices for training MLPNN, BRNN, GFFNN, SVR-L, SVR-P, SVR-S, SVR-RBF, DT, RF, LSTM, LSTM-PSO, and LSTM-MPA in the prediction of CSFAGC are presented in Table 4, and their ratings with final ranks are illustrated in Table 4. Noteworthy, the computed statistical metrics for testing developed models and ranking results are summarized in Table 5 and Table 6, respectively. As can be found, the training and testing final rating denotes that the optimized LSTM by the MPA model, with a total rate of 177 and 176 for the training and testing parts, respectively, is the most robust and accurate compared to the different AI techniques.

In the training phase, the LSTM-MPA model indicated the most accurate estimation level with Adj. R^2^ = 0.9929, MAE = 0.516, MAPE = 1.1452, NS = 0.9938, R = 0.997, R^2^ = 0.994, RMSE = 0.8332, VAF = 99.3794, WI = 0.9589, WMAPE = 0.0119, PI = 1.1535, Bias = 0.516, SI = 0.0192, p = 0.0096, and MRE = 0.0004. In the testing step, the performance indexes were determined as Adj. R^2^ = 0.9842, MAE = 0.6884, MAPE = 2.182, NS = 0.987, R = 0.9949, R^2^ = 0.9898, RMSE = 1.1893, VAF = 98.7351, WI = 0.9079, WMAPE = 0.0163, PI = 0.7822, Bias = 0.6884, SI = 0.028, p = 0.0141, and MRE = −0.0112. After comprehensively comparing the techniques, the value of other statistical indexes is presented in Table 3 and Table 6 for training and testing, respectively. The correlation plots for the developed models (MLPNN, BRNN, GFFNN, SVR-L, SVR-P, SVR-S, SVR-RBF, DT, and RF) in the training and testing stages are demonstrated in Figure 7 and Figure 8, respectively.

### 2.5. Comparison of the Proposed Model with the Literature

Providing a comparison with the various AI approaches used in the literature for CSFAGC prediction can be useful in addition to comparing the nine models used in this research to one another. Table 7 provides an overview of the majority of past studies in the period from 2017 to 2022. In Table 7, the statistical indices of R^2^ for training and testing parts are used. Table 7 also summarizes the results achieved by the nine AI models employed in our research. It can be found that the developed LSTM-MPA model presents better accuracy compared to most of the models presented in the literature as well as other developed models in this study. Noteworthy, as shown in Table 6, the model proposed by Kaveh et al. [59] addressed the most accurate estimation results with an R^2^ of 0.96 and RMSE of 3.66 by implementing the MARS model. Nevertheless, only 91 and 23 data were employed in their study to train and test parts, respectively. Other studies also used datasets with a low number of data. Nevertheless, the acceptable performance of an artificial intelligence approach is unverifiable using small amounts of data. The proposed LSTM, LSTM-PSO, and LSTM-MPA models have the most accuracy by determining the R^2^ values of 0.959, 0.976, and 0.990 and RMSE values of 2.836, 1.905, and 1.189, respectively. Herein, the LSTM-MPA model presented the highest performance level and is capable of predicting CSFAGC with the best accuracy. Therefore, the LSTM-MPA model has outperformed each developed model so far.

It should be noted that the compressive strength of concrete is not only dependent on time. It is multivariable functional, such as water-to-cement ratio (*w*/*c*), sand contents, gravel contents, and the shape and size of the samples. However, other predictive models can be constructed by importing these parameters into the new model. Therefore, the accuracy of the proposed models should be compared to these models. Ali et al. [110] used new parameters in their models. They prepared 420 data samples involving several inputs such as water/cement ratio (*w*/*c*) ranging between 0.1 and 1, cement content (C) ranged between 153.81 and 1200 kg/m^3^, fine aggregate content (FA) ranged between 492 and 2270 kg/m^3^, coarse aggregate content (CA) ranged between 617 and 2900 kg/m^3^, superplasticizer (SP) ranged between 0% and 6.7%, coarse aggregate size (CAS) ranged between 6 and 50.8 mm, fine aggregate size (FAS) ranged between 0.025 and 10 mm, nanosilica content (NS%) ranged between 0% and 15%, *w*/*c* of 0.4–0.6, and the curing time (days) ranged between 3 and 180. They developed linear regression (LR), multilinear regression (MLR), nonlinear regression (NLR), pure quadratic (PQ), interaction (IA), and full quadratic (FQ) models for predicting the compressive strength of the concrete modified with various nanosilica contents. The FQ model is a new predictive model with a high level of accuracy that they first presented. Their FQ model was the better predictive approach that yielded an R^2^ of 0.96 and an RMSE of 3.49 MP. It can be concluded that our LSTM-MPA model outperformed compared to the model presented by Ali et al. [110]. Furthermore, the effectiveness of shapes and sizes of specimens of recycled concrete aggregate in estimating long-term behavior varying compressive strength ranges was studied by Ibrahim et al. [111]. They applied several soft computing techniques involving LR, NLR, FQ, and pure quadratic (PQ) models. Noteworthy, they investigated their model performance in various ranges of compressive strength, i.e., 80–20, 20–55, and 55–80 MPa. Their model performance has excellent performance in the range of 55–80 MPa for LR, PQ, and FQ models. However, the best performance of their model was related to the range of 20–55 MPa (very good) for the NLR model. We attempt to compare their results with our results in various ranges of compressive strength. Based on this, we organized Table 8 to show how our models perform in various ranges. As can be seen in Table 8, the best performance of all models is relevant to the compressive strength range of 55–74 MPa. In addition, the performance of the LSTM-MPA model is superior in each of the three ranges. It can be concluded that our proposed model is capable of predicting CSFAGC at the maximum level of accuracy. Hence, the LSTM-MPA is a reliable model for predicting the compressive strength of concrete.

**Figure 9:**
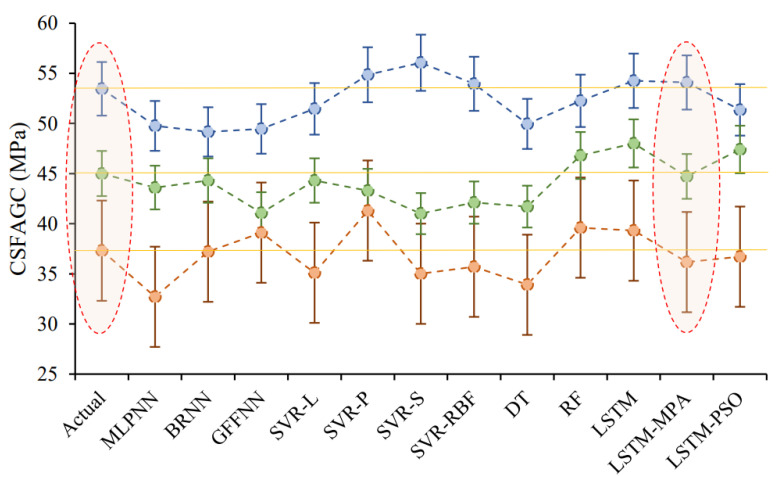
Error bar of developed models for three datasets for the same testing condition.

For more comparison, an error bar was designed, as depicted in Figure 9. This figure shows the ±5% error for three datasets for the same testing condition. The parameter values for these three datasets were as follows: SiO_2_ of 51.19%, Na_2_O of 2.12%, CaO of 5.57%, Al_2_O_3_ of 24%, Fe_2_O_3_ of 6.6%, FA of 400 kg/m^3^, Cagg of 950 kg/m^3^, Fagg of 850 kg/m^3^, SH of 57 kg/m^3^, SS of 143 kg/m^3^, EW of 40–60 kg/m^3^, SP of 28 kg/m^3^, SH concentration of 12 Molarity, percentage of SiO_2_ in SS of 29.43%, percentage of Na_2_O in SS of 14.26%, curing time of 24 h, and curing temperature of 70 °C. As can be seen in Figure 9, the result of the proposed MPA-LSTM is very close to the actual values of CSFAGC.

### 2.6. Sensitivity Analysis

In this study, the LSTM-MPA model (the best model) was investigated to specify the sensitivity by removing one of the input parameters from the prediction framework (in turn) and analyzing its effect on the CSFAGC estimation performance concerning RMSE and R^2^. Herein, various combinations of the test dataset are utilized to determine the most influential inputs. The sensitive results are reported in Table 9. To find important performance-decisive parameters, the R^2^ and RMSE of each combination were determined, each of them was scored separately, and their summation was calculated. Finally, each combination was prioritized in descending order. This technique has been successfully applied by researchers [112,113,114,115].

Consequently, the combination with the highest rate is assigned the lowest rank, and vice versa. To determine the most effective parameter schematically, the score of each combination is colored red. The combination with the highest amount of R^2^ and RMSE, as well as the lowest score, has more intensity of color. In addition, the combination with the lowest total score and rank has more color intensity. Therefore, as can be seen in Table 9, curing time (R^2^ = 0.951, RMSE = 6.189), Fe_2_O_3_ (R^2^ = 0.942, RMSE = 3.589), SH (R^2^ = 0.956, RMSE = 4.789), CaO (R^2^ = 0.942, RMSE = 3.189), Na_2_O (R^2^ = 0.952, RMSE = 4.489), and SH concentration (R^2^ = 0.950, RMSE = 3.403) were the most influential performance-decisive inputs for LSTM-MPA CSFAGC estimating.

## 3. Summary and Conclusions

This study focuses on developing a robust model for estimating CSFAGC on the basis of the chemic combinations of the geopolymer concrete components, mainly FA and sodium silicate. A collection of main papers from 2000 to 2020 was considered sufficient for the collection of the required datasets. Three main innovations of this study are using the LSTM model for predicting FAGC, optimizing the LSTM model by a new evolutionary algorithm of marine predators algorithm (MPA), and considering the six new inputs in the modeling process, such as aggregate to total mass ratio, fine aggregate to total aggregate mass ratio, FASiO_2_:Al_2_O_3_ molar ratio, FA SiO_2_:Fe_2_O_3_ molar ratio, AA Na_2_O: SiO_2_ molar ratio, and the sum of SiO_2_, Al_2_O_3_, and Fe_2_O_3_ percent in FA. The LSTM technique was applied to model and forecast the relationship between effective parameters and CSFAGC. Furthermore, the hyperparameters of the LSTM model were optimized by two metaheuristic algorithms, i.e., PSO and MPA. The hybrid LSTM-PSO and LSTM-MPA were developed to improve prediction results and enhance the performance capacity of the LSTM model. Furthermore, the performance of developed models is compared to other artificial intelligence methods such as MLPNN, BRNN, GFFNN, SVR, RF, and DT. The main results of this study are summarized as follows:

A comparison between the developed LSTM technique in current research and the LSTM-PSO and LSTM-MPA models shows that the manually adjusted LSTM model presents lower accuracy. In fact, adjusting the hyperparameters of the LSTM model by the PSO and MPA models increases performance and decreases computational time. The obtained results indicate that the LSTM-MPA with Adj. R^2^ = 0.9929, MAE = 0.516, MAPE = 1.1452, NS = 0.9938, R = 0.997, R^2^ = 0.994, RMSE = 0.8332, VAF = 99.3794, WI = 0.9589, WMAPE = 0.0119, PI = 1.1535, Bias = 0.516, SI = 0.0192, p = 0.0096, and MRE = 0.0004 in the training step and Adj. R^2^ = 0.9842, MAE = 0.6884, MAPE = 2.182, NS = 0.987, R = 0.9949, R^2^ = 0.9898, RMSE = 1.1893, VAF = 98.7351, WI = 0.9079, WMAPE = 0.0163, PI = 0.7822, Bias = 0.6884, SI = 0.028, p = 0.0141, and MRE = −0.0112 in the testing step outperformance compared to LSTM and LSTM-PSO. Therefore, the proposed LSTM-MPA structure can successfully estimate the CSFAGC.

The performance capacity of LSTM-MPA was evaluated with other artificial intelligence models. The results indicate that the R^2^ and RMSE values for models were, respectively, MLPNN (R^2^ = 0.896, RMSE = 3.745), BRNN (R^2^ = 0.931, RMSE = 2.785), GFFNN (R^2^ = 0.926, RMSE = 2.926), SVR-L (R^2^ = 0.921, RMSE = 3.017), SVR-P (R^2^ = 0.920, RMSE = 3.291), SVR-S (R^2^ = 0.934, RMSE = 2.823), SVR-RBF (R^2^ = 0.916, RMSE = 3.114), DT (R^2^ = 0.934, RMSE = 2.711), and RF (R^2^ = 0.938, RMSE = 2.892). Hence, the LSTM-MPA capabilities were noticed and recognized as the superior model for predicting CSFAGC.

In the end, the effectiveness of input parameters on the output was determined by implementing a sensitivity analysis. The results showed that the most effective performance-decisive inputs for LSTM-MPA CSFAGC estimating were curing time, Fe_2_O_3_, and SH.

## 4. Methods

### 4.1. Long Short-Term Memory Network (LSTM)

**Figure 10:**
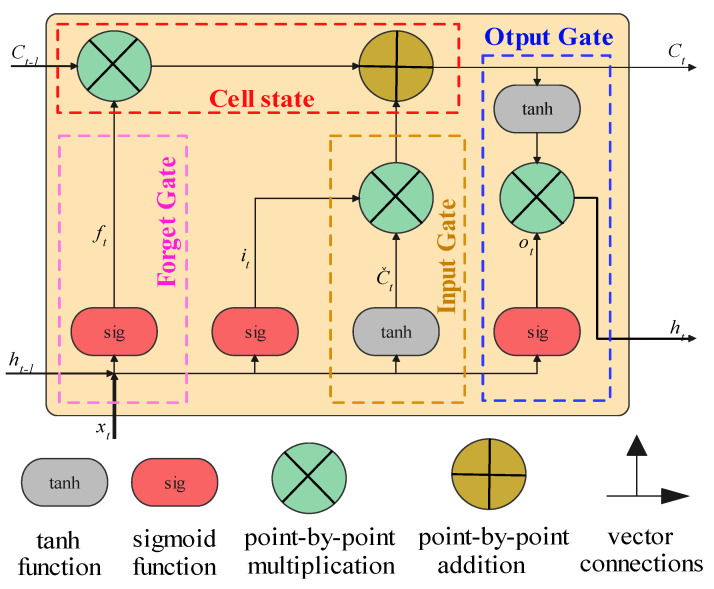
The general structure of the LSTM model.

A long short-term memory network (LSTM) is a state-of-the-art technique for analyzing long sequences of data in time-series-based analyses. The LSTM is known as the variation in recurrent neural networks (RNNs), which substitutes a memory cell for a neuron [116]. The LSTM depends on three separate gates to maintain and modify its cell state: an input gate (*i_t_*), an output gate (*o_t_*), and a forget gate (*f_t_*) (Figure 10). The quantity of dataset that must be removed from the current cell is determined by the forget gate. The output(s) gate determines how much information the current cell can output, while the input gate manages how much information must be encoded into the cell. Since the LSTM’s gated network allows information to be retained over a large number of time steps, it is able to resolve the vanishing gradient issue that affects the majority of RNN-based models. An LSTM memory cell’s calculating procedure may be described mathematically as follows [117]:

(1) $$f_{t}=\sigma \left(W_{f}x_{t}+U_{f}h_{t-1}+b_{f}\right)$$

(2) $$i_{t}=\sigma \left(W_{i}x_{i}+U_{i}h_{t-1}+b_{i}\right)$$

(3) $$o_{t}=\sigma \left(W_{o}x_{t}+U_{o}h_{t-1}+b_{o}\right)$$

(4) $$c_{t}=\left(f\times c_{t-1}\right)+\left(i_{t}\times \sigma_{c}\right)\times \left(W_{c}x_{t}+U_{c}h_{t-1}+b_{c}\right)$$

(5) $$h_{t}=o_{t}\times tanh\left(c_{t}\right)$$

where the input is *x_t_*, the result of the LSTM network is *h_t_*, and the cell state is represented by *c_t_*, *σ*; *tanh* are the sigmoid and hyperbolic tangent activation functions, *b_o_*, *b_i_*, *b_f_*, and *b_c_* represent the structure biases, and *W_f_*, *W_i_*, *W_o_*, and *W_c_* stand for the weights for the forget, input, and output gates as well as cell state, respectively.

### 4.2. Particle Swarm Optimization (PSO)

A population-based metaheuristic approach called particle swarm optimization iteratively proceeds to discover the best individual particle by optimizing a problem. In actuality, it is appropriate for very large-scale problems and makes very few assumptions about the current problem [118,119,120]. Positions are updated during PSO construction when better positions are discovered using a specific merit function. Kennedy and Eberhart presented PSO as a population-based optimization technique [121,122,123]. Particles are positioned into an N-dimensional search region in a PSO approach that uses an evolutionary computational algorithm. To find a good spot, PSO moves particles across the search region. This approach uses a particle swarm to find the ideal location. The following equations may be used to calculate the location and velocity of a particle based on its motion [124,125]:

(6) $$V_{n}=w\times V+C_{1}\times r_{1}\times \left(p_{b}-X\right)+C_{2}\times r_{2}\times \left(g_{b}-X\right)$$

(7) $$X_{n}=X\times V_{n}$$

where *X*, *V*, *X_new_*, and *V_new_* stand for the current position, the current velocity, the new position, and the new velocity, respectively; *C_1_* and *C_2_* indicate learning factors; w for inertial weights; and *r_1_* and *r_2_* are random values from the range [0, 1] [126,127,128].

### 4.3. Marine Predators Algorithm (MPA)

Natural predators are the inspiration for the marine predator algorithm, and an optimum predator stands as a solution. Constantly changing throughout the process are the peculiarities of the hunt. When the fitness of the hunt exceeds that of the predator, the predator supplants the hunt. In this way, the eddy effects in the ocean and the Fish Aggregating Device (FAD) impact are incorporated to prevent premature convergence into local optimums [129,130]. The explanation of the MPA technique is described as follows:

(1) Initiate the elite and prey matrices in order to produce the starting individuals. In the following, this algorithm is shown mathematically [129]:
  (8) $$U_{0}=U_{min}+rand\left(U_{max}-U_{min}\right)$$

The variable rand lies within [0, 1], where *U_max_* and *U_min_* represent upper and lower bounds, respectively.

According to the average number of repetitions, the predation process may be broken down into three phases. The motion rate of the hunt is greater than that of the predators at the beginning and during the first third of the stage. During this time, the prey demonstrates Brownian movement while the location of the predator stays unchanged. The following is a mathematical explanation [129]:

(9) $$D=\left|C\times {\vec{X}}_{p}\left(t\right)-{\vec{X}}_{p}\left(t\right)\right|$$

(10) $$\vec{X}\left(t+1\right)={\vec{X}}_{p}\left(t\right)-\vec{A}\times D$$

(11) $$\vec{A}=2\vec{a}\times {\vec{r}}_{1}-\vec{a}$$

(12) $$\vec{C}=2\times {\vec{r}}_{2}$$

where ${\vec{R}}_{B}$ is the Brownian motion-generated random vector; ${\vec{S}}_{i}$ denotes the step vector; Elitei is a top-tier predator assemblage known as a matrix; Preyi represents the prey matrix; ⊗ denotes the operator for term-by-term multiplications; P indicates a constant ranging inside [0, 1]. In this particular piece of research, we will be using the value 0.5; ${\vec{R}}_{i}$ stands for a random vector whose elements will all be evenly distributed within the range inside [0, 1]; and n will be the number of the population.

The population is split in two during the second stage, with the first half being in charge of exploration and the second half being in charge of exploitation. The predator’s movement speed is now equivalent to that of the prey. Following is the mathematical descriptor [129]:

(13) $$\left\{\begin{array}{c} {\vec{S}}_{1}={\vec{R}}_{L}⊗\left[Elite_{i}-{\vec{R}}_{L}⊗Prey_{i}\right]\\ Prey_{i}=Prey_{i}+\left(P\times {\vec{R}}_{i}⊗{\vec{S}}_{i}\right) \end{array}\right.$$

(14) $$\left\{\begin{array}{c} {\vec{S}}_{1}={\vec{R}}_{L}⊗\left[{\vec{R}}_{L}⊗Prey_{i}-Elite_{i}\right]\\ Prey_{i}=Elite_{i}+\left(P\times CF⊗{\vec{S}}_{i}\right)\\ CF={\left(1-\frac{Iter}{Max_{Iter}}\right)}^{\left(\frac{2Iter}{Max_{Iter}}\right)} \end{array}\right.$$

Iter is the current iteration, whereas *Max_Iter_* is the maximum number of iterations. ${\vec{R}}_{L}$ is a random vector produced by Levy motion.

During the final stage, predators move faster than prey, which results in an optimum motion by the predators known as the Levy motion. Mathematically, this can be expressed as follows [129]:

(15) $$\left\{\begin{array}{c} {\vec{S}}_{1}={\vec{R}}_{L}⊗\left[Elite_{i}-{\vec{R}}_{L}⊗Prey_{i}\right]\\ Prey_{i}=Elite_{i}+\left(P\times {\vec{R}}_{i}⊗{\vec{S}}_{i}\right) \end{array}\right.$$

(2) The occurrence of slipping into a local optimum may be successfully avoided by the implementation of the eddy running and the FADs effects, and the mathematical representation of this condition is as follows [129]:
  (16) $$Prey_{i}=Prey_{i}+CF\left[U_{min}+{\vec{R}}_{L}⊗\left(U_{max}-U_{min}\right)\right]⊗Kr\leq FADs$$
  (17) $$Prey_{i}+\left[FADs\left(1-r\right)\right]\left(Prey_{r1}-Prey_{r2}\right)r>FADs$$
  where FADs stand for the impact parameter, which is set to 0.18 in our study, K denotes the random binary vector, *r* indicates a uniform random integer between 0 and 1, and *r*_1_ and *r*_2_ denote the random hunt in the hunt matrix, respectively.

### 4.4. Data Description

The research papers that were published between the years 2000 and 2020 were reviewed to collect the dataset for developing models. As previously noted, in order to execute the improved LSTM model utilizing metaheuristic algorithms, the predictor models for forecasting the CSFAGC should be developed. This association was developed in this article using a hybrid machine learning method, namely LSTM. There are 17 input variables and 1 target parameter in the database. The inputs include 17 variables that affect the CSFAG, such as percentage of SiO_2_ (SiO_2_), percentage of Na_2_O (Na_2_O), percentage of CaO (CaO), percentage of Al_2_O_3_ (Al_2_O_3_), percentage of Fe_2_O_3_ (Fe_2_O_3_), FA, coarse aggregate (CAgg), fine aggregate (FAgg), SH, SS, extra water (EW), superplasticizer (SP), SH concentration, percentage of SiO_2_ in SS, percentage of Na_2_O in SS, curing time, curing temperature, and CS.

The criteria used to determine the chemical composition of FA were the SiO_2_:Al_2_O_3_ molar ratio and the summation of Al_2_O_3_, Fe_2_O_3_, and SiO_2_% in FA. Moreover, the SiO_2_/Fe_2_O_3_ molar ratio was taken into consideration as a crucial variable due to the component of the geo-polymerization process in which Fe_2_O_3_ is involved. One of the crucial components of FAGC is the AA-to-FA mass ratio. Therefore, in the current paper, the Al_2_O_3_ in FA:SiO_2_ in AA molar ratio, the summation of SiO_2_ and Na_2_O in AA to the quantity of SiO_2_, Al_2_O_3_, and Fe_2_O_3_ in FA mass ratio, and the summation of SiO_2_ and Na_2_O in AA to the value of SiO_2_ and Al_2_O_3_ in FA mass ratio were used as options to express these parameters. The chemical components of AA that play a role in the geo-polymerization processes are represented by the denominator in the second and third examples. Additionally, the fractions’ denominators show which chemical specifications of the FA are involved in the geo-polymerization processes. To ascertain the role of Fe_2_O_3_ in the reaction processes, the difference between these denominators is assessed [131,132,133].

In lieu of the SS:SH mass ratio, the SH concentration and chemical composition of SS, AA, and H_2_O:Na_2_O include a Na_2_O:SiO_2_ molar ratio. The water-to-FA mass ratio could be specified using the mentioned rates as well as other AA:FA ratios, as mentioned before. The impact of the H_2_O:Na_2_O molar ratio on the practicality of FAGC is the other justification for the selection. Furthermore, the described ratio shows a considerable impact on the CSFAGC [9]. Two key parameters on the CSFAGC are the curing temperature and the superplasticizer-to-FA mass ratio, which are used in the development of the LSTM predictive model. These 17 parameters serve as the prerequisites and motivating elements for the prediction of CSFAGC. Furthermore, CSFAGC is mostly predicted using these parameters by both machine learning algorithms and empirical equations.

**Figure 11:**
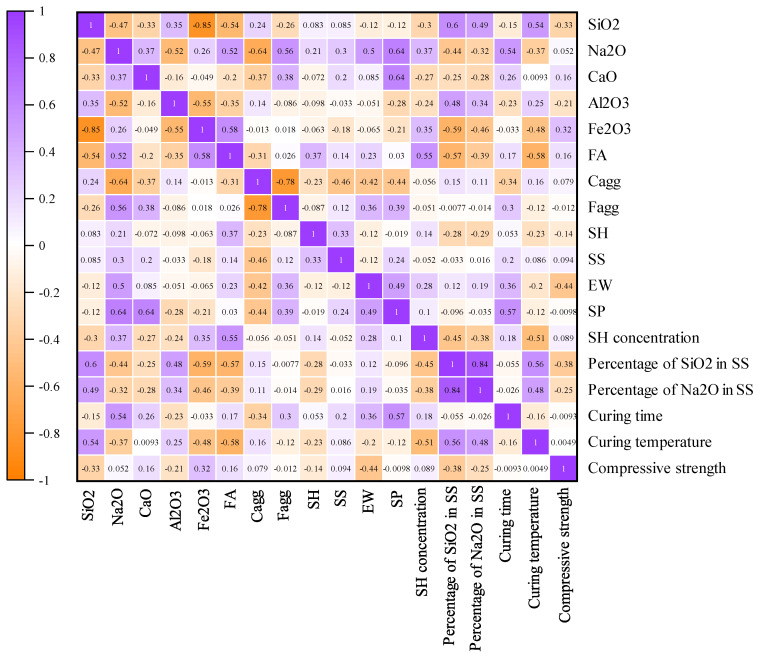
Pearson correlation coefficients between inputs and CS.

**Figure 12:**
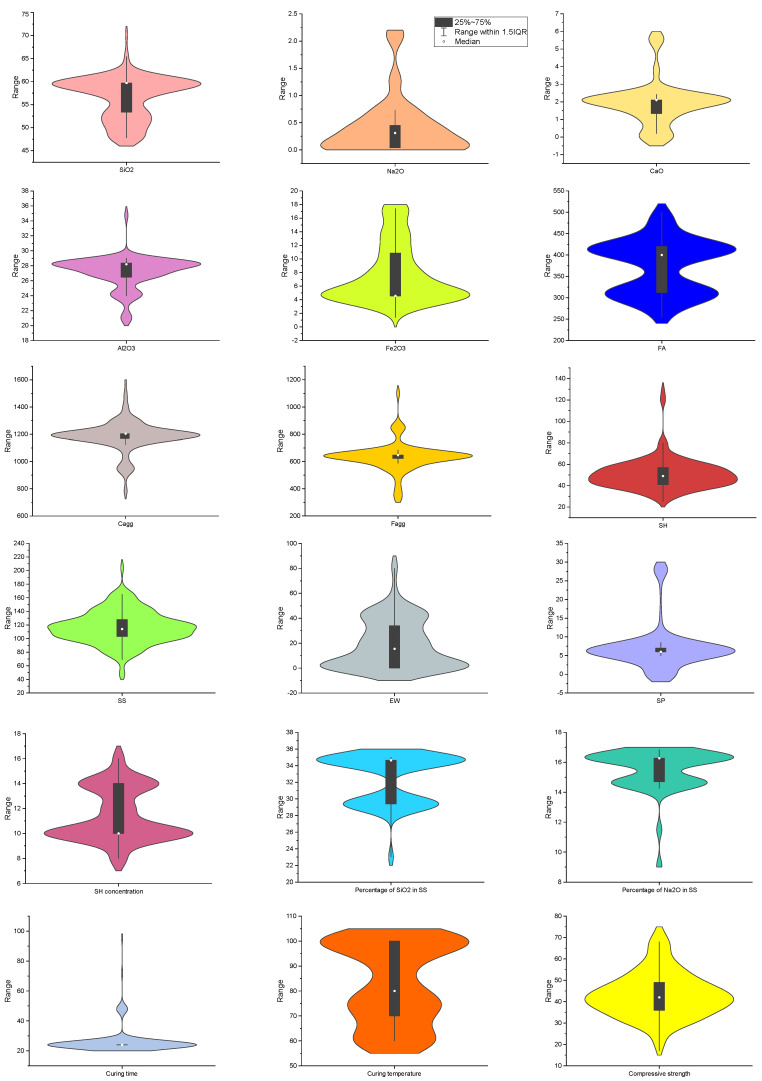
Input parameters and their violin plot.

Table 10 summarizes the descriptive statistics of the abovementioned parameters. For more data analysis, the Pearson correlation coefficients between 17 input parameters and CSFAGC are determined by using Equation (18) [134,135,136,137], with the obtained Pearson correlation coefficients depicted in Figure 11. The figure demonstrates the level at which CSFAGC establishes correlations with the inputs. It can be seen that CSFAGC shows the maximum negative correlation (−0.44) with EW. Figure 12 exhibits the violin plot of parameters.

(18) $$r=\frac{\sum_{i=1}^{n}\left(\left(X_{i}-\bar{X}\right)\times \left(Y_{i}-\bar{Y}\right)\right)}{\left(\sqrt{\sum_{i=1}^{n}{\left(X_{i}-\bar{X}\right)}^{2}}\right)\times \left(\sqrt{\sum_{i=1}^{n}{\left(Y_{i}-\bar{Y}\right)}^{2}}\right)}$$

where *r* is the Pearson coefficient, and n indicates the number of the data [138].

### 4.5. Model Validation and Evaluation

**Figure 13:**
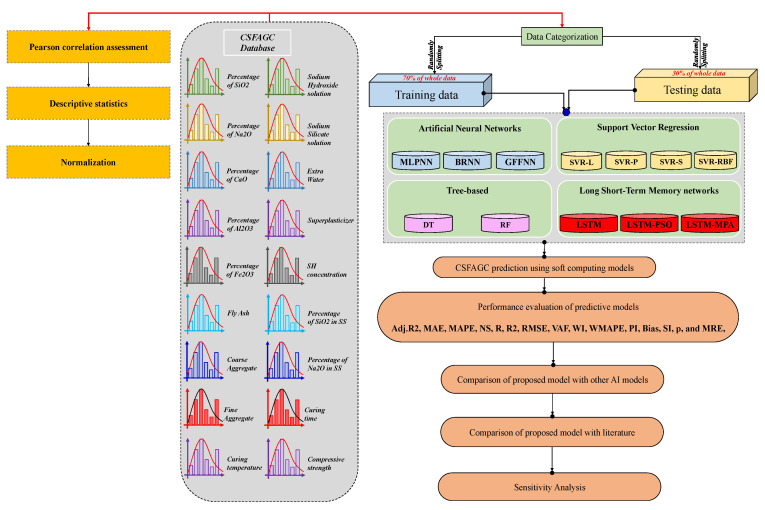
Flowchart of the methodology of research.

The flowchart of this study is revealed in Figure 13. Three metaheuristic hybrid models involving LSTM-TSA, LSTM-PSO, and LSTM-MPA have been developed to model and predict the CSFAGC. In this regard, the required pre-analysis should be performed.

Data normalization is recognized as an essential pre-analysis in data sciences and machine learning. To eliminate the dimensionality implications of the parameters, data normalization should be conducted at the pre-analysis step. As a result, before model development, the inputs and output parameters are standardized in the range of (0,1) using Equation (19), which is introduced as the “min–max” normalization method [138,139,140]:

(19) $$x_{n}=\frac{x_{i}-x_{1}}{x_{2}-x_{1}}$$

in which *x*_1_ is the minimum and *x*_2_ denotes the maximum. *n* is the number of data, and *x_n_* denotes normalized values.

After the normalization process, the whole available dataset is split into train (Tr) and test (Ts) portions. Based on this fact, 70% (113 samples) of the whole dataset are allocated as the training part in a random process; however, 30% of the remaining 42 samples are distinguished as the test sets based on previous studies [138,139,140,141,142]. Also, training data were applied to train the developed model, and test data were applied to analyze the performance of the models [143,144]. Accordingly, 14 statistical indices, including Mean Absolute Error (MAE), Weighted Mean Absolute Percentage Error (WMAPE), Coefficient of Determination (R^2^), Adjusted R^2^ (adj. R^2^), Nash–Sutcliffe efficiency (NS), Mean Relative Error (MRE), Willmott’s Index of agreement (WI), Performance Index (PI), root mean square error (RMSE), Mean Absolute Percentage Error (MAPE), and Variance Account For (VAF), BIAS, SI, and ρ for evaluating the capacity of constructed models were determined. These performance evaluation indicators are calculated as follows [27,143,144,145,146,147,148,149,150,151,152]:

(20) $$Adj.\;R^{2}=1-\frac{\left(n-1\right)}{\left(n-p-1\right)}\left(1-R^{2}\right)$$

(21) $$MAE=\frac{1}{n}\sum_{i=1}^{m}\left|\left(P_{i}-O_{i}\right)\right|$$

(22) $$MAPE=\frac{1}{n}\sum_{i=1}^{m}\left|\frac{O_{i}-P_{i}}{O_{i}}\right|\times 100$$

(23) $$NS=1-\frac{\sum_{i=1}^{m}{\left(O_{i}-P_{i}\right)}^{2}}{\sum_{i=1}^{m}{\left(O_{i}-\bar{O}\right)}^{2}}$$

(24) $$R^{2}=\frac{\sum_{i=1}^{m}{\left(O_{i}-\bar{O}\right)}^{2}-\sum_{i=1}^{m}{\left(O_{i}-P_{i}\right)}^{2}}{\sum_{i=1}^{m}{\left(O_{i}-\bar{O}\right)}^{2}}$$

(25) $$RMSE=\sqrt{\frac{1}{n}\sum_{i=1}^{m}{\left(O_{i}-P_{i}\right)}^{2}}$$

(26) $$VAF=100\times \left(1-\frac{var\left(O_{i}-P_{i}\right)}{var\left(O_{i}\right)}\right)$$

(27) $$WI=1-\left(\frac{\sum_{i=1}^{m}{\left(O_{i}-P_{i}\right)}^{2}}{\sum_{i=1}^{m}{\left(\left|P_{i}-\bar{O}\right|+\left|O_{i}-\bar{O}\right|\right)}^{2}}\right)$$

(28) $$WMAPE=\frac{\sum_{i=1}^{m}\left(\left|\frac{O_{i}-P_{i}}{O_{i}}\right|\times O_{i}\right)}{\sum_{i=1}^{m}O_{i}}$$

(29) $$MRE=\frac{1}{n}\sum_{i=1}^{n}\left(\frac{O_{i}-P_{i}}{O_{i}}\right)$$

(30) $$PI=Adj.\;R^{2}+0.01\;VAF-RMSE$$

(31) $$Bias=\frac{1}{n}\sum_{i=1}^{n}\left(P_{i}-O_{i}\right)$$

(32) $$SI=\frac{RMSE}{\frac{1}{n}\sum_{i=1}^{n}P_{i}}$$

(33) $$\rho =\frac{SI}{1+R}$$

where *p* is the number of independent parameters and *n* is the number of datasets. Noteworthy, *y_i_*, ${\hat{y}}_{i}$ and $\bar{y}$ signify the experimental, predicted, and average values of experimental data, respectively [147,153,154].

R^2^, adj. R^2^, VAF, PI, WI, and NS are examples of statistical indices for monitoring trends. Moreover, statistical indices for calculating errors are RMSE, MAE, MRE, WMAPE, MAPE, BIAS, SI, and *ρ*. Nevertheless, the error-specifying metric quantifies the prediction mode error, and the parameters in the first category show the trends of the predictive model [155,156]. In the field of statistical sciences, R^2^ is used to measure the linear correlations between measured and predicted values, VAF is employed to determine the relative variance, WI is utilized to explain the level of estimation errors, NS assesses the relative size of the remnant in comparison to the real variance of the dataset, and PI is applied for measuring the combined effects of the adj. R^2^, VAF, and RMSE. When comparing a collection of forecasts that assess precision, RMSE and MAE are the typical indicators. Although MAE quantifies average error magnitude, MAPE computes average error percentage, and WMAPE calculates the sum of errors weighted by the real outputs, RMSE reflects the root mean square discrepancies between the measured and predicted values. These indices can be used to estimate the model’s error and accuracy level. Nevertheless, a superior model has higher R^2^, VAF, PI, and NS and lower values of RMSE, MAE, MAPE, WMAPE, BIAS, SI, and ρ.

### 4.6. Development of Proposed Model

The dataset is incorporated into the LSTM model at this stage, and the starting settings model for its hyperparameters is taken into account. Each predictive model has a particular set of these hyperparameters. Actually, determining optimum values or types of hyperparameters is highly time and labor-intensive [157,158,159]. In the present study, the hyperparameters of LSTMs are tuned using a new optimization algorithm named the marine predators algorithm (MPA). There are three phases to both optimizing the LSTM hyperparameters and combining optimization algorithms with the LSTM technique:

(1) The MPA is utilized to optimize the hyperparameters (including the number of epochs, number of hidden neurons, batch sizes, dropout rates, and activation functions), as well as to determine their initial values in a general model for the regression of the LSTM.
(2) After training the model with input datasets using the LSTM model from the preceding phase and the hyperparameters’ initial values, Bayesian theory is used to optimize the hyperparameters.
(3) Following the initial metaheuristic algorithm process, the new dataset is imported into the LSTM model to obtain the final estimation result. The initial values of the hyperparameters have been optimized to enable immediate implementation of the optimization problems and prevent the parameter values from quickly falling into an optimal local value.

Next, through evaluation indices, i.e., Equations (15)–(30), the optimized model’s performance is investigated. The model’s hyperparameters are now adjusted once more until it performs at its best. The strongest optimized model is lastly regarded as the best model.

Comparison between the expected and actual results allows for evaluating the predictive performance of the optimized model.

## Figures and Tables

**Table 1:** Literature details employing artificial intelligence methods.

No.	References	Year	Type of AI	Material Used
1	[57]	2017	ANN	FA
2	[58]	2017	ANN	FA
3	[59]	2018	M5, MARS	FA
4	[60]	2018	RKSA	FA
5	[61]	2018	ANFIS	-
6	[62]	2018	ANN	-
7	[63]	2019	GEP	-
8	[64]	2019	GEP	Natural Zeolite
9	[65]	2019	ANN	FA, Silica fume, ground granulated blast furnace slag, Rice husk ash
10	[66]	2019	SVM	FA
11	[67]	2019	RF	FA, ground granulated blast furnace slag
	[68]	2020	Experimental	-
	[69]	2020	SVM	FA
12	[70]	2020	SVM	FA
13	[71]	2020	GEP	ground granulated blast furnace slag
14	[72]	2020	GEP	-
	[73]	2020	GEP	-
15	[64]	2020	RF, GEP	-
16	[74]	2020	MR	Crumb rubber with Silica fume
17	[44]	2020	GEP	-
18	[75]	2020	ANFIS	Palm oil fuel ash
19	[76]	2020	RSM, GEP	Steel Fibers
20	[77]	2021	SVM	FA
21	[78]	2021	DEA	FA
22	[79]	2021	GEP, ANN, DT	FA
23	[80]	2021	GEP, DT and Bagging	FA
24	[81]	2021	Bagging, boosting, and ANNs	FA
25	[82]	2021	DT, ANN, BR, GB	FA
26	[83]	2021	MARS, ANN	-

SVM: support vector machine, GEP: gene expression programming, DEA: data envelopment analysis, ANNs: artificial neural networks, DT: decision tree, RF: random forest, RSM: response surface method, MARS: multivariate adaptive regression spline, RKSA: random kitchen sink algorithm, ANFIS: adaptive neuro-fuzzy inference system, MR: multivariate regression.

**Table 2:** Review of the studies that use a large number of parameters.

Author	References	Year	Number of Parameters	Journal
Pavithra et al.	[17]	2016	17	Journal of Cleaner Production
Toufigh et al.	[87]	2021	17	Construction and Building Materials
Farhan et al.	[88]	2019	17	Construction and Building Materials
Wardhono et al.	[89]	2017	17	Construction and Building Materials
Lokuge et al.	[90]	2018	17	Construction and Building Materials
Tanyildizi	[91]	2021	14	Cement and Concrete Composites
Ahmed et al.	[92]	2011	17	International Journal of Civil & Environmental Engineering
Hardjito and Rangan	[14]	2005	17	-
Hardjito et al.	[93]	2005	17	Australian Journal of Structural Engineering
Olivia and Nikraz	[94]	2012	17	Materials & Design
Sarker et al.	[95]	2013	17	Materials & Design
Sujatha et al.	[96]	2012	17	Asian Journal of Civil Engineering
Sumajouw and Rangan	[97]	2006	17	-
Vora and Dave	[98]	2013	17	Procedia Engineering
Gunasekara et al.	[99]	2021	14	Polymers

**Table 3:** The obtained statistical indices for the training phase of models.

	MLPNN	BRNN	GFFNN	SVR-L	SVR-P	SVR-S	SVR-RBF	DT	RF	LSTM	LSTM-MPA	LSTM-PSO
Adj. R^2^	0.8898	0.9253	0.9100	0.9184	0.9099	0.9281	0.9234	0.9358	0.9443	0.9676	0.9929	0.9768
MAE	2.9177	2.3558	2.6265	2.5912	2.7150	2.3177	2.4469	2.2000	2.0584	1.5212	0.5160	1.3177
MAPE	7.0599	5.7984	6.4777	6.3411	6.4540	5.6829	6.0216	5.4565	4.9885	3.7969	1.1452	3.1460
NS	0.8973	0.9315	0.9214	0.9182	0.9161	0.9334	0.9259	0.9386	0.9476	0.9715	0.9938	0.9792
R	0.9521	0.9678	0.9611	0.9648	0.9610	0.9690	0.9670	0.9724	0.9761	0.9862	0.9970	0.9901
R^2^	0.9065	0.9366	0.9236	0.9308	0.9236	0.9390	0.9351	0.9455	0.9528	0.9725	0.9940	0.9804
RMSE	3.3821	2.7629	2.9596	3.0195	3.0576	2.7239	2.8737	2.6156	2.4161	1.7816	0.8332	1.5221
VAF	89.9107	93.1533	92.1569	91.8628	91.8108	93.4083	92.7618	93.8820	95.1852	97.1730	99.3794	97.9285
WI	0.3244	0.5533	0.4713	0.4715	0.4483	0.5649	0.5173	0.6031	0.6530	0.8123	0.9589	0.8647
WMAPE	0.0670	0.0541	0.0603	0.0595	0.0624	0.0532	0.0562	0.0505	0.0473	0.0349	0.0119	0.0303
PI	−1.5932	−0.9061	−1.1281	−1.1825	−1.2296	−0.8617	−1.0226	−0.7410	−0.5199	0.1577	1.1535	0.4340
Bias	2.9177	2.3558	2.6265	2.5912	2.7150	2.3177	2.4469	2.2000	2.0584	1.5212	0.5160	1.3177
SI	0.0785	0.0633	0.0678	0.0690	0.0710	0.0630	0.0667	0.0603	0.0564	0.0411	0.0192	0.0350
*p*	0.0402	0.0322	0.0345	0.0351	0.0362	0.0320	0.0339	0.0306	0.0285	0.0207	0.0096	0.0176
MRE	0.0091	−0.0020	−0.0054	−0.0042	0.0114	0.0073	0.0120	0.0050	0.0152	0.0041	0.0004	0.0029

**Table 4:** Rating statistical indices of training models.

	MLPNN	BRNN	GFFNN	SVR-L	SVR-P	SVR-S	SVR-RBF	DT	RF	LSTM	LSTM-MPA	LSTM-PSO
Adj. R^2^	1	6	3	4	2	7	5	8	9	10	12	11
MAE	1	6	3	4	2	7	5	8	9	10	12	11
MAPE	1	6	2	4	3	7	5	8	9	10	12	11
NS	1	6	4	3	2	7	5	8	9	10	12	11
R	1	6	3	4	2	7	5	8	9	10	12	11
R^2^	1	6	3	4	2	7	5	8	9	10	12	11
RMSE	1	6	4	3	2	7	5	8	9	10	12	11
VAF	1	6	4	3	2	7	5	8	9	10	12	11
WI	1	6	3	4	2	7	5	8	9	10	12	11
WMAPE	1	6	3	4	2	7	5	8	9	10	12	11
PI	1	6	4	3	2	7	5	8	9	10	12	11
Bias	1	6	3	4	2	7	5	8	9	10	12	11
SI	1	6	4	3	2	7	5	8	9	10	12	11
*p*	1	6	4	3	2	7	5	8	9	10	12	11
MRE	4	10	12	11	3	5	2	6	1	7	9	8
Total Rate	18	94	59	61	32	103	72	118	127	147	177	162
Rank	12	7	10	9	11	6	8	5	4	3	1	2

**Table 5:** The obtained statistical indices for the testing phase of models.

	MLPNN	BRNN	GFFNN	SVR-L	SVR-P	SVR-S	SVR-RBF	DT	RF	LSTM	LSTM-MPA	LSTM-PSO
Adj. R^2^	0.8389	0.8930	0.8860	0.8771	0.8763	0.8979	0.8706	0.8972	0.9039	0.9358	0.9842	0.9624
MAE	3.2224	2.4837	2.5351	2.7347	2.9388	2.4673	2.7265	2.3020	2.5020	2.3735	0.6884	1.6816
MAPE	8.2233	6.2646	6.4209	6.8691	7.7863	6.5515	7.0134	5.6864	6.5376	5.8742	2.1820	4.2857
NS	0.8714	0.9289	0.9215	0.9166	0.9007	0.9270	0.9111	0.9326	0.9233	0.9263	0.9870	0.9667
R	0.9465	0.9648	0.9625	0.9595	0.9592	0.9665	0.9573	0.9662	0.9685	0.9791	0.9949	0.9878
R^2^	0.8959	0.9309	0.9264	0.9206	0.9201	0.9341	0.9164	0.9336	0.9379	0.9586	0.9898	0.9757
RMSE	3.7452	2.7853	2.9256	3.0166	3.2907	2.8225	3.1138	2.7110	2.8922	2.8357	1.1893	1.9049
VAF	87.8520	93.0403	92.1633	92.0645	90.5636	92.7396	91.1511	93.2660	92.4074	95.3553	98.7351	97.5173
WI	0.1404	0.4959	0.4615	0.4123	0.3412	0.5028	0.3716	0.5131	0.4919	0.5100	0.9079	0.7671
WMAPE	0.0763	0.0588	0.0600	0.0648	0.0696	0.0584	0.0646	0.0545	0.0593	0.0562	0.0163	0.0398
PI	−2.0278	−0.9619	−1.1179	−1.2188	−1.5088	−0.9972	−1.3317	−0.8811	−1.0642	−0.9463	0.7822	0.0327
Bias	3.2224	2.4837	2.5351	2.7347	2.9388	2.4673	2.7265	2.3020	2.5020	2.3735	0.6884	1.6816
SI	0.0869	0.0666	0.0691	0.0726	0.0766	0.0665	0.0741	0.0643	0.0690	0.0645	0.0280	0.0441
*p*	0.0446	0.0339	0.0352	0.0370	0.0391	0.0338	0.0379	0.0327	0.0350	0.0326	0.0141	0.0222
MRE	−0.0222	0.0055	−0.0019	0.0097	−0.0194	−0.0047	0.0025	−0.0019	0.0132	−0.0410	−0.0112	−0.0242

**Table 6:** Rating statistical indices of testing models.

	MLPNN	BRNN	GFFNN	SVR-L	SVR-P	SVR-S	SVR-RBF	DT	RF	LSTM	LSTM-MPA	LSTM-PSO
Adj. R^2^	1	6	5	4	3	8	2	7	9	10	12	11
MAE	1	7	5	3	2	8	4	10	6	9	12	11
MAPE	1	8	7	4	2	5	3	10	6	9	12	11
NS	1	9	5	4	2	8	3	10	6	7	12	11
R	1	6	5	4	3	8	2	7	9	10	12	11
R^2^	1	6	5	4	3	8	2	7	9	10	12	11
RMSE	1	9	5	4	2	8	3	10	6	7	12	11
VAF	1	8	5	4	2	7	3	9	6	10	12	11
WI	1	7	5	4	2	8	3	10	6	9	12	11
WMAPE	1	7	5	3	2	8	4	10	6	9	12	11
PI	1	8	5	4	2	7	3	10	6	9	12	11
Bias	1	7	5	3	2	8	4	10	6	9	12	11
SI	1	7	5	4	2	8	3	10	6	9	12	11
*p*	1	7	5	4	2	8	3	9	6	10	12	11
MRE	10	3	6	2	9	7	4	5	1	12	8	11
Total Rate	24	105	78	55	40	114	46	134	94	139	176	165
Rank	12	6	8	9	11	5	10	4	7	3	1	2

**Table 7:** Performance comparison of developed models with the literature.

No.	References		Year	Type of ML	Train Data	Test Data	R^2^	RMSE	Rate of R^2^	Rate of RMSE	Toral Rate	Rank
1	[57]	64	2017	ANN	91	23	0.95	-	19	-	19	9
2	[58]	65	2017	ANN			0.66	17.22	1	1	2	24
3	[59]	61	2018	M5’	91	23	0.94	4.39	18	5	23	6
				MARS			0.96	3.66	22	9	31	1
4	[60]	62	2018	RKSA	32	8	-	0.046	-	11	11	14
6	[63]	51	2019	GEP	242	61	0.928	-	16	-	16	10
				ANN			0.895		9	-	9	19
				PSO-ANN			0.91		12	-	12	13
7	[64]	57	2019	RF	-	-	0.96	-	22	-	22	7
				DT			0.899		10	-	10	17
				ANN			0.89		7	-	7	20
				GEP			0.9		11	-	11	14
8	[65]	58	2019	ANN	113	28	0.966	-	24	-	24	5
10	[67]	60	2019	RF	-	-	0.954	3.977	20	7	27	3
11	[69]	54	2020	SVR-L	-	-	0.798	6.173	3	2	5	21
				SVR-P			0.891	4.619	8	3	11	14
				SVR-S			0.889	4.5	6	4	10	17
				SVR-RBF			0.939	3.38	17	10	27	3
12	[70]	55	2020	SVM	92	23	0.955	3.783	21	8	29	2
13	[71]	56	2020	GEP	-	-	0.923	4.238	15	6	21	8
14	[72]	44	2020	GEP	251	53	0.914	-	14	-	14	11
22	[79]	53	2021	GEP	-	-	0.885	-	5	-	5	21
				ANN			0.85		4	-	4	23
				DT			0.719		2	-	2	24
23	[80]	66	2021	Bagging	-	-	0.911	-	13	-	13	12
This study				MLPNN	113	42	0.896	3.745	-	-	-	-
				BRNN			0.931	2.785	-	-	-	-
				GFFNN			0.926	2.926	-	-	-	-
				SVR-L			0.921	3.017	-	-	-	-
				SVR-P			0.920	3.291	-	-	-	-
				SVR-S			0.934	2.823	-	-	-	-
				SVR-RBF			0.916	3.114	-	-	-	-
				DT			0.934	2.711	-	-	-	-
				RF			0.938	2.892	-	-	-	-
				LSTM			0.959	2.836	-	-	-	-
				LSTM-PSO			0.976	1.905	-	-	-	-
				LSTM-MPA			0.990	1.189	-	-	-	-

**Table 8:** Model performance in compressive strength estimation of various ranges.

Models	Compressive Strength Ranges (MPa)	No. of Data	R^2^	RMSE	Model Performance Rank
MLPNN	17–25	5	0.99931	3.2075	2
	25–55	274	0.795053	3.5218	3
	55–74	60	0.999508	3.1178	1
BRNN	17–25	5	0.999387	2.4702	2
	25–55	274	0.866674	2.6984	3
	55–74	60	0.99961	2.6838	1
GFFNN	17–25	5	0.998907	3.2175	2
	25–55	274	0.853496	2.8934	3
	55–74	60	0.999561	2.8624	1
SVR-L	17–25	5	0.999174	3.2234	2
	25–55	274	0.85187	2.9101	3
	55–74	60	0.999485	3.2108	1
SVR-P	17–25	5	0.998944	3.5771	2
	25–55	274	0.852225	2.9641	3
	55–74	60	0.999401	3.394	1
SVR-S	17–25	5	0.998881	3.3749	2
	25–55	274	0.871657	2.8079	3
	55–74	60	0.999735	2.2369	1
SVR-RBF	17–25	5	0.998896	3.3205	2
	25–55	274	0.865197	2.948	3
	55–74	60	0.999603	2.7671	1
DT	17–25	5	0.998952	3.1777	2
	25–55	274	0.874379	2.6727	3
	55–74	60	0.999683	2.514	1
RF	17–25	5	0.999019	3.4563	2
	25–55	274	0.896183	2.4396	3
	55–74	60	0.999667	2.4925	1
LSTM	17–25	5	0.999691	1.7499	2
	25–55	274	0.932546	1.9588	3
	55–74	60	0.999784	2.058	1
LSTM-MPA	17–25	5	0.999756	3.1315	2
	25–55	274	0.996983	0.4632	3
	55–74	60	0.999846	1.8441	1
LSTM-PSO	17–25	5	0.999841	1.4332	2
	25–55	274	0.957039	1.5363	3
	55–74	60	0.999868	1.6667	1

**Table 9:** Sensitivity analysis of the LSTM-MPA technique for the testing phase.

Combination of Input																		LSTM-MPA		Rating R^2^ and RMSE		Total Rate	Rank
x_1_	x_2_	x_3_	x_4_	x_5_	x_6_	x_7_	x_8_	x_9_	x_10_	x_11_	x_12_	x_13_	x_14_	x_15_	x_16_	x_17_	x_18_	R^2^	RMSE	R^2^	RMSE		
																		0.990	1.189	1	1	2	19
																		0.979	4.589	2	17	19	10
																		0.952	4.489	12	15	27	5
																		0.942	3.189	18	10	28	4
																		0.945	2.789	16	8	24	7
																		0.942	3.589	18	13	31	2
																		0.959	1.689	9	2	11	17
																		0.945	2.689	16	6	22	8
																		0.965	4.499	6	16	22	8
																		0.956	4.789	11	18	29	3
																		0.960	2.789	8	8	16	13
																		0.964	2.689	7	6	13	16
																		0.957	2.239	10	4	14	15
																		0.950	3.403	14	12	26	6
																		0.973	2.189	3	3	6	18
																		0.969	4.115	4	14	18	12
																		0.951	6.189	13	19	32	1
																		0.966	3.189	5	10	15	14
																		0.950	2.325	14	5	19	10

x_1_: SiO_2_, x_2_: Na_2_O, x3: CaO, x_4_: Al_2_O_3_, x_5_: Fe_2_O_3_, x6: FA, x_7_: Cagg, x_8_: Fagg, x_9_: SH, x_10_: SS, x_11_: EW, x_12_: SP, x_13_: SH concentration, x_14_: Percentage of SiO_2_ in SS, x_15_: Percentage of Na_2_O in SS, x_16_: Curing time, x_17_: Curing temperature, and x_18_: Compressive strength.

**Table 10:** Descriptive statistics of the related parameters.

Parameters	Symbol	Unit	Mean	Median	StD	Kurtosis	Skewness	Min	Max
* Percentage of SiO_2_	SiO_2_	%	56.49	59.70	4.59	−0.46	−0.50	47.80	70.30
* Percentage of Na_2_O	Na_2_O	%	0.41	0.31	0.54	3.71	2.02	0.04	2.12
* Percentage of CaO	CaO	%	1.95	2.10	1.23	2.69	1.12	0.03	5.57
* Percentage of Al_2_O_3_	Al_2_O_3_	%	27.14	28.21	2.09	3.17	−0.78	20.70	34.75
* Percentage of Fe_2_O_3_	Fe_2_O_3_	%	7.77	4.57	4.29	−0.12	1.08	1.40	17.40
Fly Ash	FA	kg/m^3^	372.68	400.00	62.81	−1.13	−0.06	255.00	500.00
Coarse Aggregate	CAgg	kg/m^3^	1186.12	1204.00	109.03	3.71	−0.59	785.00	1591.00
Fine Aggregate	FAgg	kg/m^3^	633.26	647.00	107.65	5.27	0.51	318.00	1100.00
Sodium Hydroxide solution	SH	kg/m^3^	50.63	49.00	14.46	10.22	2.40	25.00	129.00
Sodium Silicate solution	SS	kg/m^3^	116.38	114.00	23.70	0.96	0.26	48.00	204.00
Extra Water	EW	kg/m^3^	19.03	15.50	19.61	0.26	0.90	0.00	86.00
Superplasticizer	SP	kg/m^3^	7.09	6.00	6.14	6.16	2.40	0.00	28.00
SH concentration	SH	Molarity	11.45	10.00	2.09	−1.00	0.47	8.00	16.00
Percentage of SiO_2_ in SS	-	%	32.46	34.64	2.90	−0.10	−0.83	23.00	35.01
Percentage of Na_2_O in SS	-	%	15.52	16.27	1.37	8.12	−2.37	9.10	16.84
Curing time	-	h	26.37	24.00	8.96	27.26	4.74	24.00	96.00
Curing temperature	-	^°^C	82.93	80.00	16.04	−1.55	−0.19	60.00	100.00
Compressive strength	CS	MPa	43.14	42.00	10.57	0.39	0.52	17.00	74.00

* Chemical composition of FA. SiO_2_: silicon dioxide, Na_2_O: sodium oxide, CaO: calcium oxide, Al_2_O_3_: aluminum oxide, and Fe_2_O_3_: ferric oxide.

## Data Availability

The data presented in this study are openly available in article.

## References

[1] Masoud M.A., El-Khayatt A.M., Mahmoud K.A., Rashad A.M., Shahien M.G., Bakhit B.R., Zayed A.M. (2023). Valorization of Hazardous Chrysotile by H_3_BO_3_ Incorporation to Produce an Innovative Eco-Friendly Radiation Shielding Concrete: Implications on Physico-Mechanical, Hydration, Microstructural, and Shielding Properties. Cem. Concr. Compos..

[2] Masoud M.A., Rashad A.M., Sakr K., Shahien M.G., Zayed A.M. (2020). Possibility of Using Different Types of Egyptian Serpentine as Fine and Coarse Aggregates for Concrete Production. Mater. Struct..

[3] Masoud M.A., El-Khayatt A.M., Kansouh W.A., Sakr K., Shahien M.G., Zayed A.M. (2020). Insights into the Effect of the Mineralogical Composition of Serpentine Aggregates on the Radiation Attenuation Properties of Their Concretes. Constr. Build. Mater..

[4] Zayed A.M., Masoud M.A., Rashad A.M., El-Khayatt A.M., Sakr K., Kansouh W.A., Shahien M.G. (2020). Influence of Heavyweight Aggregates on the Physico-Mechanical and Radiation Attenuation Properties of Serpentine-Based Concrete. Constr. Build. Mater..

[5] Zayed A.M., Masoud M.A., Shahien M.G., Gökçe H.S., Sakr K., Kansouh W.A., El-Khayatt A.M. (2021). Physical, Mechanical, and Radiation Attenuation Properties of Serpentine Concrete Containing Boric Acid. Constr. Build. Mater..

[6] Masoud M.A., Kansouh W.A., Shahien M.G., Sakr K., Rashad A.M., Zayed A.M. (2020). An Experimental Investigation on the Effects of Barite/Hematite on the Radiation Shielding Properties of Serpentine Concretes. Prog. Nucl. Energy.

[7] Hendriks C.A., Worrell E., De Jager D., Blok K., Riemer P. Emission Reduction of Greenhouse Gases from the Cement Industry. Proceedings of the Fourth International Conference on Greenhouse Gas Control Technologies.

[8] Hansen J., Johnson D., Lacis A., Lebedeff S., Lee P., Rind D., Russell G. (1981). Climate Impact of Increasing Atmospheric Carbon Dioxide. Science.

[9] Resketi N.A., Toufigh V. (2019). Enhancement of Brick-Mortar Shear Bond Strength Using Environmental Friendly Mortars. Constr. Build. Mater..

[10] Kosarimovahhed M., Toufigh V. (2020). Sustainable Usage of Waste Materials as Stabilizer in Rammed Earth Structures. J. Clean. Prod..

[11] McLellan B.C., Williams R.P., Lay J., Van Riessen A., Corder G.D. (2011). Costs and Carbon Emissions for Geopolymer Pastes in Comparison to Ordinary Portland Cement. J. Clean. Prod..

[12] Davidovits J. (1991). Geopolymers: Inorganic Polymeric New Materials. J. Therm. Anal. Calorim..

[13] Kong D.L.Y., Sanjayan J.G. (2010). Effect of Elevated Temperatures on Geopolymer Paste, Mortar and Concrete. Cem. Concr. Res..

[14] Hardjito D., Rangan B.V. (2005). Development and Properties of Low-Calcium Fly Ash-Based Geopolymer Concrete.

[15] Liu J.D., Li G.C., Yang S., Huang J.D. (2020). Prediction Models for Evaluating the Strength of Cemented Paste Backfill: A Comparative Study. Minerals.

[16] Huang J.D., Zhang Y., Sun Y.T., Ren J.L., Zhao Z.D., Zhang J.F. (2021). Evaluation of Pore Size Distribution and Permeability Reduction Behavior in Pervious Concrete. Constr. Build. Mater..

[17] Pavithra P., Reddy M.S., Dinakar P., Rao B.H., Satpathy B.K., Mohanty A.N. (2016). A Mix Design Procedure for Geopolymer Concrete with Fly Ash. J. Clean. Prod..

[18] Al Bakri A.M.M., Kamarudin H., Bnhussain M., Rafiza A., Zarina Y. (2010). Effect of Na_2_SiO_3_/NaOH Ratios and NaOH Molarities on Compressive Strength of Fly Ash Based Geopolymer Cement. Green Concr. UniMAP Sch. Mater. Eng..

[19] Phoo-ngernkham T., Maegawa A., Mishima N., Hatanaka S., Chindaprasirt P. (2015). Effects of Sodium Hydroxide and Sodium Silicate Solutions on Compressive and Shear Bond Strengths of FA–GBFS Geopolymer. Constr. Build. Mater..

[20] Joseph B., Mathew G. (2012). Influence of Aggregate Content on the Behavior of Fly Ash Based Geopolymer Concrete. Sci. Iran..

[21] Abdulkareem O.A., Ramli M. (2015). Optimization of Alkaline Activator Mixing and Curing Conditions for a Fly Ash-Based Geopolymer Paste System. Mod. Appl. Sci..

[22] Tang Z., Hu Y., Tam V.W.Y., Li W. (2019). Uniaxial Compressive Behaviors of Fly Ash/Slag-Based Geopolymeric Concrete with Recycled Aggregates. Cem. Concr. Compos..

[23] Ma C.-K., Awang A.Z., Omar W. (2018). Structural and Material Performance of Geopolymer Concrete: A Review. Constr. Build. Mater..

[24] Assi L.N., Deaver E.E., Ziehl P. (2018). Effect of Source and Particle Size Distribution on the Mechanical and Microstructural Properties of Fly Ash-Based Geopolymer Concrete. Constr. Build. Mater..

[25] De Silva P., Sagoe-Crenstil K., Sirivivatnanon V. (2007). Kinetics of Geopolymerization: Role of Al_2_O_3_ and SiO_2_. Cem. Concr. Res..

[26] Davidovits J., Davidovits R. (2020). Ferro-Sialate Geopolymers (-Fe-O-Si-O-Al-O-).

[27] Zhou J., Su Z., Hosseini S., Tian Q., Lu Y., Luo H., Xu X., Chen C., Huang J. (2024). Decision Tree Models for the Estimation of Geo-Polymer Concrete Compressive Strength. Math. Biosci. Eng..

[28] Huang J.D., Kumar G.S., Sun Y.T. (2021). Evaluation of Workability and Mechanical Properties of Asphalt Binder and Mixture Modified with Waste Toner. Constr. Build. Mater..

[29] Huang J.D., Kumar G.S., Ren J.L., Sun Y.T., Li Y.J., Wang C.G. (2022). Towards the Potential Usage of Eggshell Powder as Bio-Modifier for Asphalt Binder and Mixture: Workability and Mechanical Properties. Int. J. Pavement Eng..

[30] (2019). A Standard Specification for Coal Fly Ash and Raw or Calcined Natural Pozzolan for Use in Concrete.

[31] Huang J.D., Sun Y.T., Zhang J.F. (2022). Reduction of Computational Error by Optimizing SVR Kernel Coefficients to Simulate Concrete Compressive Strength through the Use of a Human Learning Optimization Algorithm. Eng. Comput..

[32] Huang J.D., Asteris P.G., Pasha S.M.K., Mohammed A.S., Hasanipanah M. (2022). A New Auto-Tuning Model for Predicting the Rock Fragmentation: A Cat Swarm Optimization Algorithm. Eng. Comput..

[33] Tennakoon C., Nazari A., Sanjayan J.G., Sagoe-Crentsil K. (2014). Distribution of Oxides in Fly Ash Controls Strength Evolution of Geopolymers. Constr. Build. Mater..

[34] Khedmati M., Alanazi H., Kim Y.-R., Nsengiyumva G., Moussavi S. (2018). Effects of Na2O/SiO2 Molar Ratio on Properties of Aggregate-Paste Interphase in Fly Ash-Based Geopolymer Mixtures through Multiscale Measurements. Constr. Build. Mater..

[35] De Vargas A.S., Dal Molin D.C.C., Vilela A.C.F., Da Silva F.J., Pavao B., Veit H. (2011). The Effects of Na_2_O/SiO_2_ Molar Ratio, Curing Temperature and Age on Compressive Strength, Morphology and Microstructure of Alkali-Activated Fly Ash-Based Geopolymers. Cem. Concr. Compos..

[36] Ascensão G., Marchi M., Segata M., Faleschini F., Pontikes Y. (2020). Reaction Kinetics and Structural Analysis of Alkali Activated Fe–Si–Ca Rich Materials. J. Clean. Prod..

[37] Cui Y., Wang D., Wang Y., Sun R., Rui Y. (2019). Effects of the n (H_2_O: Na_2_Oeq) Ratio on the Geopolymerization Process and Microstructures of Fly Ash-Based Geopolymers. J. Non-Cryst. Solids.

[38] Song H., Ahmad A., Farooq F., Ostrowski K.A., Maślak M., Czarnecki S., Aslam F. (2021). Predicting the Compressive Strength of Concrete with Fly Ash Admixture Using Machine Learning Algorithms. Constr. Build. Mater..

[39] Farooq F., Czarnecki S., Niewiadomski P., Aslam F., Alabduljabbar H., Ostrowski K.A., Śliwa-Wieczorek K., Nowobilski T., Malazdrewicz S. (2021). A Comparative Study for the Prediction of the Compressive Strength of Self-Compacting Concrete Modified with Fly Ash. Materials.

[40] Khan M.A., Memon S.A., Farooq F., Javed M.F., Aslam F., Alyousef R. (2021). Compressive Strength of Fly-Ash-Based Geopolymer Concrete by Gene Expression Programming and Random Forest. Adv. Civ. Eng..

[41] Ilyas I., Zafar A., Javed M.F., Farooq F., Aslam F., Musarat M.A., Vatin N.I. (2021). Forecasting Strength of CFRP Confined Concrete Using Multi Expression Programming. Materials.

[42] Farooq F., Nasir Amin M., Khan K., Rehan Sadiq M., Javed M.F., Aslam F., Alyousef R. (2020). A Comparative Study of Random Forest and Genetic Engineering Programming for the Prediction of Compressive Strength of High Strength Concrete (HSC). Appl. Sci..

[43] Raza F., Alshameri B., Jamil S.M. (2021). Assessment of Triple Bottom Line of Sustainability for Geotechnical Projects. Environ. Dev. Sustain..

[44] Javed M.F., Farooq F., Memon S.A., Akbar A., Khan M.A., Aslam F., Alyousef R., Alabduljabbar H., Rehman S.K.U. (2020). New Prediction Model for the Ultimate Axial Capacity of Concrete-Filled Steel Tubes: An Evolutionary Approach. Crystals.

[45] Nafees A., Javed M.F., Khan S., Nazir K., Farooq F., Aslam F., Musarat M.A., Vatin N.I. (2021). Predictive Modeling of Mechanical Properties of Silica Fume-Based Green Concrete Using Artificial Intelligence Approaches: MLPNN, ANFIS, and GEP. Materials.

[46] Asteris P.G., Lourenço P.B., Roussis P.C., Adami C.E., Armaghani D.J., Cavaleri L., Chalioris C.E., Hajihassani M., Lemonis M.E., Mohammed A.S. (2022). Revealing the Nature of Metakaolin-Based Concrete Materials Using Artificial Intelligence Techniques. Constr. Build. Mater..

[47] Barkhordari M.S., Armaghani D.J., Mohammed A.S., Ulrikh D.V. (2022). Data-Driven Compressive Strength Prediction of Fly Ash Concrete Using Ensemble Learner Algorithms. Buildings.

[48] Liao J., Asteris P.G., Cavaleri L., Mohammed A.S., Lemonis M.E., Tsoukalas M.Z., Skentou A.D., Maraveas C., Koopialipoor M., Armaghani D.J. (2021). Novel Fuzzy-Based Optimization Approaches for the Prediction of Ultimate Axial Load of Circular Concrete-Filled Steel Tubes. Buildings.

[49] Biswas R., Bardhan A., Samui P., Rai B., Nayak S., Armaghani D.J. (2021). Efficient Soft Computing Techniques for the Prediction of Compressive Strength of Geopolymer Concrete. Comput. Concr..

[50] Apostolopoulou M., Asteris P.G., Armaghani D.J., Douvika M.G., Lourenço P.B., Cavaleri L., Bakolas A., Moropoulou A. (2020). Mapping and Holistic Design of Natural Hydraulic Lime Mortars. Cem. Concr. Res..

[51] Koopialipoor M., Asteris P.G., Salih Mohammed A., Alexakis D.E., Mamou A., Armaghani D.J. (2022). Introducing Stacking Machine Learning Approaches for the Prediction of Rock Deformation. Transp. Geotech..

[52] Armaghani D.J., Asteris P.G. (2021). A Comparative Study of ANN and ANFIS Models for the Prediction of Cement-Based Mortar Materials Compressive Strength. Neural Comput. Appl..

[53] Iqbal M.F., Liu Q.F., Azim I., Zhu X., Yang J., Javed M.F., Rauf M. (2020). Prediction of Mechanical Properties of Green Concrete Incorporating Waste Foundry Sand Based on Gene Expression Programming. J. Hazard. Mater..

[54] Golafshani E.M., Behnood A. (2021). Predicting the Mechanical Properties of Sustainable Concrete Containing Waste Foundry Sand Using Multi-Objective ANN Approach. Constr. Build. Mater..

[55] Sun Y., Hanhan I., Sangid M.D., Lin G. (2020). Predicting Mechanical Properties from Microstructure Images in Fiber-Reinforced Polymers Using Convolutional Neural Networks. arXiv preprint.

[56] Kabiru O.A., Owolabi T.O., Ssennoga T., Olatunji S.O. (2014). Performance Comparison of SVM and ANN in Predicting Compressive Strength of Concrete. J. Comput. Eng..

[57] Belalia Douma O., Boukhatem B., Ghrici M., Tagnit-Hamou A. (2017). Prediction of Properties of Self-Compacting Concrete Containing Fly Ash Using Artificial Neural Network. Neural Comput. Appl..

[58] Abu Yaman M., Abd Elaty M., Taman M. (2017). Predicting the Ingredients of Self Compacting Concrete Using Artificial Neural Network. Alex. Eng. J..

[59] Kaveh A., Bakhshpoori T., Hamze-Ziabari S.M. (2018). M5’ and Mars Based Prediction Models for Properties of Selfcompacting Concrete Containing Fly Ash. Period. Polytech. Civ. Eng..

[60] Sathyan D., Anand K.B., Prakash A.J., Premjith B. (2018). Modeling the Fresh and Hardened Stage Properties of Self-Compacting Concrete Using Random Kitchen Sink Algorithm. Int. J. Concr. Struct. Mater..

[61] Vakhshouri B., Nejadi S. (2018). Prediction of Compressive Strength of Self-Compacting Concrete by ANFIS Models. Neurocomputing.

[62] Naderpour H., Rafiean A.H., Fakharian P. (2018). Compressive Strength Prediction of Environmentally Friendly Concrete Using Artificial Neural Networks. J. Build. Eng..

[63] Sarir P., Chen J., Asteris P.G., Armaghani D.J., Tahir M.M. (2021). Developing GEP Tree-Based, Neuro-Swarm, and Whale Optimization Models for Evaluation of Bearing Capacity of Concrete-Filled Steel Tube Columns. Eng. Comput..

[64] Huang J., Zhou M., Zhang J., Ren J., Vatin N.I., Sabri M.M.S. (2022). Development of a new stacking model to evaluate the strength parameters of concrete samples in laboratory. Iran. J. Sci. Technol. Trans. Civ. Eng..

[65] Asteris P.G., Kolovos K.G. (2019). Self-Compacting Concrete Strength Prediction Using Surrogate Models. Neural Comput. Appl..

[66] Selvaraj S., Sivaraman S. (2019). Prediction Model for Optimized Self-Compacting Concrete with Fly Ash Using Response Surface Method Based on Fuzzy Classification. Neural Comput. Appl..

[67] Zhang J., Ma G., Huang Y., Sun J., Aslani F., Nener B. (2019). Modelling Uniaxial Compressive Strength of Lightweight Self-Compacting Concrete Using Random Forest Regression. Constr. Build. Mater..

[68] Prachasaree W., Limkatanyu S., Hawa A., Sukontasukkul P., Chindaprasirt P. (2020). Manuscript Title: Development of Strength Prediction Models for Fly Ash Based Geopolymer Concrete. J. Build. Eng..

[69] Azimi-Pour M., Eskandari-Naddaf H., Pakzad A. (2020). Linear and Non-Linear SVM Prediction for Fresh Properties and Compressive Strength of High Volume Fly Ash Self-Compacting Concrete. Constr. Build. Mater..

[70] Saha P., Debnath P., Thomas P. (2020). Prediction of Fresh and Hardened Properties of Self-Compacting Concrete Using Support Vector Regression Approach. Neural Comput. Appl..

[71] Shahmansouri A.A., Akbarzadeh Bengar H., Jahani E. (2019). Predicting Compressive Strength and Electrical Resistivity of Eco-Friendly Concrete Containing Natural Zeolite via GEP Algorithm. Constr. Build. Mater..

[72] Aslam F., Farooq F., Amin M.N., Khan K., Waheed A., Akbar A., Javed M.F., Alyousef R., Alabdulijabbar H. (2020). Applications of Gene Expression Programming for Estimating Compressive Strength of High-Strength Concrete. Adv. Civ. Eng..

[73] Shahmansouri A.A., Akbarzadeh Bengar H., Ghanbari S. (2020). Compressive Strength Prediction of Eco-Efficient GGBS-Based Geopolymer Concrete Using GEP Method. J. Build. Eng..

[74] Buši R. (2020). Prediction Models for the Mechanical Properties of Self-Compacting Concrete with Recycled Rubber and Silica Fume. Materials.

[75] Al-mughanam T., Aldhyani T.H.H., Alsubari B., Al-yaari M. (2020). Modeling of Compressive Strength of Sustainable Self-Compacting Concrete Incorporating Treated Palm Oil Fuel Ash Using Artificial Neural Network. Sustainability.

[76] Nematzadeh M., Shahmansouri A.A., Fakoor M. (2020). Post-Fire Compressive Strength of Recycled PET Aggregate Concrete Reinforced with Steel Fibers: Optimization and Prediction via RSM and GEP. Constr. Build. Mater..

[77] Huang J., Zhou M., Zhang J., Ren J., Vatin N.I., Sabri M.M.S. (2022). The use of GA and PSO in evaluating the shear strength of steel fiber reinforced concrete beams. KSCE J. Civ. Eng..

[78] Balf F.R., Kordkheili H.M., Kordkheili A.M. (2021). A New Method for Predicting the Ingredients of Self-Compacting Concrete (SCC) Including Fly Ash (FA) Using Data Envelopment Analysis (DEA). Arab. J. Sci. Eng..

[79] Ahmad A., Farooq F., Ostrowski K.A., Śliwa-Wieczorek K., Czarnecki S. (2021). Application of Novel Machine Learning Techniques for Predicting the Surface Chloride Concentration in Concrete Containing Waste Material. Materials.

[80] Ahmad A., Farooq F., Niewiadomski P., Ostrowski K., Akbar A., Aslam F., Alyousef R. (2021). Prediction of Compressive Strength of Fly Ash Based Concrete Using Individual and Ensemble Algorithm. Materials.

[81] Farooq F., Ahmed W., Akbar A., Aslam F., Alyousef R. (2021). Predictive Modeling for Sustainable High-Performance Concrete from Industrial Wastes: A Comparison and Optimization of Models Using Ensemble Learners. J. Clean. Prod..

[82] Ahmad A., Ostrowski K.A., Maślak M., Farooq F., Mehmood I., Nafees A. (2021). Comparative Study of Supervised Machine Learning Algorithms for Predicting the Compressive Strength of Concrete at High Temperature. Materials.

[83] Asteris P.G., Skentou A.D., Bardhan A., Samui P., Pilakoutas K. (2021). Predicting Concrete Compressive Strength Using Hybrid Ensembling of Surrogate Machine Learning Models. Cem. Concr. Res..

[84] Huang J.D., Koopialipoor M., Armaghani D.J. (2020). A Combination of Fuzzy Delphi Method and Hybrid ANN-Based Systems to Forecast Ground Vibration Resulting from Blasting. Sci. Rep..

[85] Sun Y.T., Li G.C., Zhang J.F., Huang J.D. (2021). Rockburst Intensity Evaluation by a Novel Systematic and Evolved Approach: Machine Learning Booster and Application. Bull. Eng. Geol. Environ..

[86] Ji Z., Zhou M.M., Wang Q., Huang J.D. (2024). Predicting the International Roughness Index of JPCP and CRCP Rigid Pavement: A Random Forest (RF) Model Hybridized with Modified Beetle Antennae Search (MBAS) for Higher Accuracy. CMES-Comput. Model. Eng. Sci..

[87] Toufigh V., Jafari A. (2021). Developing a Comprehensive Prediction Model for Compressive Strength of Fly Ash-Based Geopolymer Concrete (FAGC). Constr. Build. Mater..

[88] Farhan N.A., Sheikh M.N., Hadi M.N.S. (2019). Investigation of Engineering Properties of Normal and High Strength Fly Ash Based Geopolymer and Alkali-Activated Slag Concrete Compared to Ordinary Portland Cement Concrete. Constr. Build. Mater..

[89] Wardhono A., Gunasekara C., Law D.W., Setunge S. (2017). Comparison of Long Term Performance between Alkali Activated Slag and Fly Ash Geopolymer Concretes. Constr. Build. Mater..

[90] Lokuge W., Wilson A., Gunasekara C., Law D.W., Setunge S. (2018). Design of Fly Ash Geopolymer Concrete Mix Proportions Using Multivariate Adaptive Regression Spline Model. Constr. Build. Mater..

[91] Tanyildizi H. (2021). Predicting the Geopolymerization Process of Fly Ash-Based Geopolymer Using Deep Long Short-Term Memory and Machine Learning. Cem. Concr. Compos..

[92] Ahmed M.F., Nuruddin M.F., Shafiq N. (2011). Compressive Strength and Workability Characteristics of Low-Calcium Fly Ash-Based Self-Compacting Geopolymer Concrete. Int. J. Civ. Environ. Eng..

[93] Hardjito D., Wallah S.E., Sumajouw D.M.J., Rangan B.V. (2005). Fly Ash-Based Geopolymer Concrete. Aust. J. Struct. Eng..

[94] Olivia M., Nikraz H. (2012). Properties of Fly Ash Geopolymer Concrete Designed by Taguchi Method. Mater. Des. (1980–2015).

[95] Sarker P.K., Haque R., Ramgolam K. (2013). V Fracture Behaviour of Heat Cured Fly Ash Based Geopolymer Concrete. Mater. Des..

[96] Sujatha T., Kannapiran K., Nagan S. (2012). Strength Assessment of Heat Cured Geopolymer Concrete Slender Column. ASIAN J. Civ. Eng. (Build. Hous.).

[97] Sumajouw M., Rangan B.V. (2006). Low-Calcium Fly Ash-Based Geopolymer Concrete: Reinforced Beams and Columns.

[98] Vora P.R., Dave U. (2013). V Parametric Studies on Compressive Strength of Geopolymer Concrete. Procedia Eng..

[99] Gunasekara C., Atzarakis P., Lokuge W., Law D.W., Setunge S. (2021). Novel Analytical Method for Mix Design and Performance Prediction of High Calcium Fly Ash Geopolymer Concrete. Polymers.

[100] Ahmed H.U., Mohammed A.A., Mohammed A.S. (2023). Effectiveness of Nano-SiO_2_ on the Mechanical, Durability, and Microstructural Behavior of Geopolymer Concrete at Different Curing Ages. Arch. Civ. Mech. Eng..

[101] Ahmed H.U., Mohammed A.S., Faraj R.H., Abdalla A.A., Qaidi S.M.A., Sor N.H., Mohammed A.A. (2023). Innovative Modeling Techniques Including MEP, ANN and FQ to Forecast the Compressive Strength of Geopolymer Concrete Modified with Nanoparticles. Neural Comput. Appl..

[102] Qaidi S., Yahia A., Tayeh B.A., Unis H., Faraj R., Mohammed A. (2022). 3D Printed Geopolymer Composites: A Review. Mater. Today Sustain..

[103] Ahmed H.U., Mostafa R.R., Mohammed A., Sihag P., Qadir A. (2023). Support Vector Regression (SVR) and Grey Wolf Optimization (GWO) to Predict the Compressive Strength of GGBFS-Based Geopolymer Concrete. Neural Comput. Appl..

[104] Ahmed H.U., Mohammed A.A., Mohammed A. (2022). Soft Computing Models to Predict the Compressive Strength of GGBS/FA-Geopolymer Concrete. PLoS ONE.

[105] Ahmed H.U., Mohammed A.S., Mohammed A.A. (2022). Proposing Several Model Techniques Including ANN and M5P-Tree to Predict the Compressive Strength of Geopolymer Concretes Incorporated with Nano-Silica. Environ. Sci. Pollut. Res..

[106] Ahmed H.U., Mohammed A.S., Mohammed A.A., Faraj R.H. (2021). Systematic Multiscale Models to Predict the Compressive Strength of Fly Ash-Based Geopolymer Concrete at Various Mixture Proportions and Curing Regimes. PLoS ONE.

[107] Mahmoodzadeh A., Nejati H.R., Mohammadi M., Ibrahim H.H., Rashidi S., Rashid T.A. (2022). Forecasting Tunnel Boring Machine Penetration Rate Using LSTM Deep Neural Network Optimized by Grey Wolf Optimization Algorithm. Expert. Syst. Appl..

[108] Huang J.D., Duan T.H., Zhang Y., Liu J.D., Zhang J., Lei Y.W. (2020). Predicting the Permeability of Pervious Concrete Based on the Beetle Antennae Search Algorithm and Random Forest Model. Adv. Civ. Eng..

[109] Ren J.L., Xu Y.S., Zhao Z.D., Chen J.C., Cheng Y.Y., Huang J.D., Yang C.X., Wang J. (2022). Fatigue Prediction of Semi-Flexible Composite Mixture Based on Damage Evolution. Constr. Build. Mater..

[110] Ali R., Muayad M., Mohammed A.S., Asteris P.G. (2022). Analysis and Prediction of the Effect of Nanosilica on the Compressive Strength of Concrete with Different Mix Proportions and Specimen Sizes Using Various Numerical Approaches. Struct. Concr..

[111] Ibrahim A.K., Dhahir H.Y., Mohammed A.S., Omar H.A., Sedo A.H. (2023). The Effectiveness of Surrogate Models in Predicting the Long-Term Behavior of Varying Compressive Strength Ranges of Recycled Concrete Aggregate for a Variety of Shapes and Sizes of Specimens. Arch. Civ. Mech. Eng..

[112] Bakhtavar E., Hosseini S., Hewage K., Sadiq R. (2021). Air Pollution Risk Assessment Using a Hybrid Fuzzy Intelligent Probability-Based Approach: Mine Blasting Dust Impacts. Nat. Resour. Res..

[113] Bakhtavar E., Hosseini S., Hewage K., Sadiq R. (2021). Green Blasting Policy: Simultaneous Forecast of Vertical and Horizontal Distribution of Dust Emissions Using Artificial Causality-Weighted Neural Network. J. Clean. Prod..

[114] Sun Y.T., Bi R.Y., Chang Q.L., Taherdangkoo R., Zhang J.F., Sun J.B., Huang J.D., Li G.C. (2021). Stability Analysis of Roadway Groups under Multi-Mining Disturbances. Appl. Sci. Basel.

[115] Cui K., Chang J., Sabri M.M.S., Huang J.D. (2022). Toughness, Reinforcing Mechanism, and Durability of Hybrid Steel Fiber Reinforced Sulfoaluminate Cement Composites. Buildings.

[116] Ranjbar I., Toufigh V. (2022). Deep Long Short-Term Memory (LSTM) Networks for Ultrasonic-Based Distributed Damage Assessment in Concrete. Cem. Concr. Res..

[117] Zhang R., Liu Y., Sun H. (2020). Physics-Informed Multi-LSTM Networks for Metamodeling of Nonlinear Structures. Comput. Methods Appl. Mech. Eng..

[118] Wang X., Hosseini S., Jahed Armaghani D., Tonnizam Mohamad E. (2023). Data-Driven Optimized Artificial Neural Network Technique for Prediction of Flyrock Induced by Boulder Blasting. Mathematics.

[119] Zhu F., Wu X., Lu Y., Huang J. (2024). Strength Estimation and Feature Interaction of Carbon Nanotubes-Modified Concrete Using Artificial Intelligence-Based Boosting Ensembles. Buildings.

[120] Wang R., Zhang J., Lu Y., Huang J. (2024). Towards Designing Durable Sculptural Elements: Ensemble Learning in Predicting Compressive Strength of Fiber-Reinforced Nano-Silica Modified Concrete. Buildings.

[121] Kennedy J., Eberhart R. Particle Swarm Optimization. Proceedings of the ICNN’95-International Conference on Neural Networks.

[122] Huang J., Zhou M., Sabri M.M.S., Yuan H. (2022). A novel neural computing model applied to estimate the dynamic modulus (DM) of asphalt mixtures by the improved beetle antennae search. Sustainability.

[123] Ren J.L., Xu Y.S., Huang J.D., Wang Y., Jia Z.R. (2021). Gradation Optimization and Strength Mechanism of Aggregate Structure Considering Macroscopic and Mesoscopic Aggregate Mechanical Behaviour in Porous Asphalt Mixture. Constr. Build. Mater..

[124] Momeni E., Jahed Armaghani D., Hajihassani M., Mohd Amin M.F. (2015). Prediction of Uniaxial Compressive Strength of Rock Samples Using Hybrid Particle Swarm Optimization-Based Artificial Neural Networks. Meas. J. Int. Meas. Confed..

[125] Armaghani D.J., Mirzaei F., Shariati M., Trung N.T., Shariati M., Trnavac D. (2020). Hybrid Ann-Based Techniques in Predicting Cohesion of Sandy-Soil Combined with Fiber. Geomech. Eng..

[126] Zhu S.-P., Keshtegar B., Seghier M.E.A.B., Zio E., Taylan O. (2022). Hybrid and Enhanced PSO: Novel First Order Reliability Method-Based Hybrid Intelligent Approaches. Comput. Methods Appl. Mech. Eng..

[127] Zhu F., Wu X., Lu Y., Huang J. (2024). Strength Reduction Due to Acid Attack in Cement Mortar Containing Waste Eggshell and Glass: A Machine Learning-Based Modeling Study. Buildings.

[128] Kasza J., Wolfe R. (2014). Interpretation of Commonly Used Statistical Regression Models. Respirology.

[129] Faramarzi A., Heidarinejad M., Mirjalili S., Gandomi A.H. (2020). Marine Predators Algorithm: A Nature-Inspired Metaheuristic. Expert. Syst. Appl..

[130] Speiser J.L., Miller M.E., Tooze J., Ip E. (2019). A Comparison of Random Forest Variable Selection Methods for Classification Prediction Modeling. Expert. Syst. Appl..

[131] Myles A.J., Feudale R.N., Liu Y., Woody N.A., Brown S.D. (2004). An Introduction to Decision Tree Modeling. J. Chemom..

[132] Ahmed H.U., Mohammed A.S., Qaidi S.M.A., Faraj R.H., Hamah Sor N., Mohammed A.A. (2023). Compressive Strength of Geopolymer Concrete Composites: A Systematic Comprehensive Review, Analysis and Modeling. Eur. J. Environ. Civ. Eng..

[133] Gao Y., Huang J.D., Li M., Dai Z.R., Jiang R.L., Zhang J.X. (2021). Chemical Modification of Combusted Coal Gangue for U(VI) Adsorption: Towards a Waste Control by Waste Strategy. Sustainability.

[134] Hosseini S., Mousavi A., Monjezi M. (2022). Prediction of Blast-Induced Dust Emissions in Surface Mines Using Integration of Dimensional Analysis and Multivariate Regression Analysis. Arab. J. Geosci..

[135] Hosseini S., Poormirzaee R., Hajihassani M., Kalatehjari R. (2022). An ANN-Fuzzy Cognitive Map-Based Z-Number Theory to Predict Flyrock Induced by Blasting in Open-Pit Mines. Rock. Mech. Rock. Eng..

[136] Zhang H., Chang Q., Li S., Huang J.D. (2022). Determining the Efficiency of the Sponge City Construction Pilots in China Based on the DEA-Malmquist Model. Int. J. Environ. Res. Public. Health.

[137] Tian Q., Su Z.L., Fiorentini N., Zhou J., Luo H., Lu Y.J., Xu X.Q., Chen C.P., Huang J.D. (2023). Ensemble Learning Models to Predict the Compressive Strength of Geopolymer Concrete: A Comparative Study for Geopolymer Composition Design. Multiscale Multidiscip. Model. Exp. Des..

[138] Hosseini S., Poormirzaee R., Hajihassani M. (2022). Application of Reliability-Based Back-Propagation Causality-Weighted Neural Networks to Estimate Air-Overpressure Due to Mine Blasting. Eng. Appl. Artif. Intell..

[139] Hasanipanah M., Monjezi M., Shahnazar A., Jahed Armaghani D., Farazmand A. (2015). Feasibility of Indirect Determination of Blast Induced Ground Vibration Based on Support Vector Machine. Meas. J. Int. Meas. Confed..

[140] Hosseini S., Javanshir S., Sabeti H., Tahmasebizadeh P. (2023). Mathematical-Based Gene Expression Programming (GEP): A Novel Model to Predict Zinc Separation from a Bench-Scale Bioleaching Process. J. Sustain. Metall..

[141] Parsajoo M., Armaghani D.J., Mohammed A.S., Khari M., Jahandari S. (2021). Tensile Strength Prediction of Rock Material Using Non-Destructive Tests: A Comparative Intelligent Study. Transp. Geotech..

[142] Hosseini S., Mousavi A., Monjezi M., Khandelwal M. (2022). Mine-to-Crusher Policy: Planning of Mine Blasting Patterns for Environmentally Friendly and Optimum Fragmentation Using Monte Carlo Simulation-Based Multi-Objective Grey Wolf Optimization Approach. Resour. Policy.

[143] Hancock J.T., Khoshgoftaar T.M. (2020). CatBoost for Big Data: An Interdisciplinary Review. J. Big Data.

[144] Huang J., Xue J. (2022). Optimization of SVR functions for flyrock evaluation in mine blasting operations. Environ. Earth Sci..

[145] Hosseini S., Poormirzaee R., Gilani S.-O., Jiskani I.M. (2023). A Reliability-Based Rock Engineering System for Clean Blasting: Risk Analysis and Dust Emissions Forecasting. Clean. Technol. Environ. Policy.

[146] Hosseini S., Pourmirzaee R., Armaghani D.J., Sabri Sabri M.M. (2023). Prediction of Ground Vibration Due to Mine Blasting in a Surface Lead–Zinc Mine Using Machine Learning Ensemble Techniques. Sci. Rep..

[147] Hosseini S., Poormirzaee R., Hajihassani M. (2022). An Uncertainty Hybrid Model for Risk Assessment and Prediction of Blast-Induced Rock Mass Fragmentation. Int. J. Rock. Mech. Min. Sci..

[148] Zhao J., Hosseini S., Chen Q., Armaghani D.J. (2023). Super Learner Ensemble Model: A Novel Approach for Predicting Monthly Copper Price in Future. Resour. Policy.

[149] Hosseini S., Khatti J., Taiwo B.O., Fissha Y., Grover K.S., Ikeda H., Pushkarna M., Berhanu M., Ali M. (2023). Assessment of the Ground Vibration during Blasting in Mining Projects Using Different Computational Approaches. Sci. Rep..

[150] Lawal A.I., Hosseini S., Kim M., Ogunsola N.O., Kwon S. (2023). Prediction of Factor of Safety of Slopes Using Stochastically Modified ANN and Classical Methods: A Rigorous Statistical Model Selection Approach. Nat. Hazards.

[151] Wang Q., Qi J., Hosseini S., Rasekh H., Huang J. (2023). ICA-LightGBM Algorithm for Predicting Compressive Strength of Geo-Polymer Concrete. Buildings.

[152] Hosseini S., Pourmirzaee R. (2023). Green Policy for Managing Blasting Induced Dust Dispersion in Open-Pit Mines Using Probability-Based Deep Learning Algorithm. Expert. Syst. Appl..

[153] Hosseini S., Monjezi M., Bakhtavar E., Mousavi A. (2021). Prediction of Dust Emission Due to Open Pit Mine Blasting Using a Hybrid Artificial Neural Network. Nat. Resour. Res..

[154] Xu W.J., Huang X., Yang Z.J., Zhou M.M., Huang J.D. (2022). Developing Hybrid Machine Learning Models to Determine the Dynamic Modulus (E*) of Asphalt Mixtures Using Parameters in Witczak 1-40D Model: A Comparative Study. Materials.

[155] Kardani N., Bardhan A., Samui P., Nazem M., Zhou A., Armaghani D.J. (2022). A Novel Technique Based on the Improved Firefly Algorithm Coupled with Extreme Learning Machine (ELM-IFF) for Predicting the Thermal Conductivity of Soil. Eng. Comput..

[156] Hosseini S., Monjezi M., Bakhtavar E. (2022). Minimization of Blast-Induced Dust Emission Using Gene-Expression Programming and Grasshopper Optimization Algorithm: A Smart Mining Solution Based on Blasting Plan Optimization. Clean. Technol. Environ. Policy.

[157] Ren J.L., Li D., Xu Y.S., Huang J.D., Liu W. (2022). Fatigue Behaviour of Rock Asphalt Concrete Considering Moisture, High-Temperature, and Stress Level. Int. J. Pavement Eng..

[158] Ma H.X., Liu J.D., Zhang J., Huang J.D. (2021). Estimating the Compressive Strength of Cement-Based Materials with Mining Waste Using Support Vector Machine, Decision Tree, and Random Forest Models. Adv. Civ. Eng..

[159] Huang J.D., Zhang J., Ren J.L., Chen H.W. (2021). Anti-Rutting Performance of the Damping Asphalt Mixtures (DAMs) Made with a High Content of Asphalt Rubber (AR). Constr. Build. Mater..

